# Bentonites Modified with Phosphomolybdic Heteropolyacid (HPMo) for Biowaste to Biofuel Production

**DOI:** 10.3390/ma12091431

**Published:** 2019-05-02

**Authors:** Alex de Nazaré de Oliveira, Marco Aurélio Barbosa de Lima, Luíza Helena de Oliveira Pires, Moisés Rosas da Silva, Patrícia Teresa Souza da Luz, Rômulo S. Angélica, Geraldo N. da Rocha Filho, Carlos Emmerson F. da Costa, Rafael Luque, Luís Adriano Santos do Nascimento

**Affiliations:** 1 Laboratory of Catalysis and Oilchemistry, Federal University of Pará, Augusto Corrêa Street, Guamá, Belém, PA 66075-110, Brazil; alexoliveiraquimica@hotmail.com (A.d.N.d.O.); marbalim@hotmail.com (M.A.B.d.L.); lulenapires@hotmail.com (L.H.d.O.P.); moisesrosas06@hotmail.com (M.R.d.S.); narciso@ufpa.br (G.N.d.R.F.); emmerson@ufpa.br (C.E.F.d.C.); 2 Laboratory of Oils of the Amazon, Federal University of Pará, Perimetral Avenue, Guamá, Belém, PA 66075-750, Brazil; 3 Department of Exact and Technologic Sciences, Federal Ubiversity of Amapá, Rod. Juscelino Kubitschek, km 02-Jardim Marco Zero, Macapá, AP 68903-419, Brazil; 4 Federal Institute of Education, Science and Technology of Pará, Campus Belém, Avenue Almirante Barroso, Marco, Belém, PA 66093-020, Brazil; pdaluz@yahoo.com; 5 Laboratory of X-Ray Diffraction, Federal University of Pará, Augusto Corrêa Street, Guamá, Belém, PA 66075-110, Brazil; rsangelica@gmail.com; 6 Department of Organic Chemistry, Universidad de Córdoba, Ctra Nnal IV-A, Km 396, E14014 Cordoba, Spain; 7 Peoples Friendship University of Russia (RUDN University), 6 Miklukho-Maklaya str., Moscow 117198, Russia

**Keywords:** heteropolyacids, bentonite, esterification, clays, heterogeneous catalysis

## Abstract

Two bentonites from Paraíba (Northeastern Brazil) were impregnated with heteropoly phosphomolybdic H_3_PMo_12_O_40_ (HPMo). The materials produced were characterized by various techniques such as N_2_ adsorption-desorption (specific surface area, SSA), Fourier transform infrared spectroscopy (FTIR), X-ray diffraction (XRD), Thermogravimetric analysis (TGA/DTG), Scanning Electron Microscopy (SEM) equipped with Dispersive Energy X-ray spectroscopy (EDS), ultraviolet-visible spectroscopy (UV-vis), acid-base titration analysis. The catalytic activity of these materials was tested in the esterification of a waste from palm oil deodorization and the main results obtained (about 93.3% of conversion) indicated that these materials have potential to act as heterogeneous solid acid catalysts. The prepared materials exhibited satisfactory catalytic performance even after a very simple recycling process in three reuse cycles, without significant loss of their activities.

## 1. Introduction

Fossil fuels have been the most commonly employed energy sources in the generation of energy coming mainly from crude oil. Due to limited and non-renewable supply of this fuel and the increase in the price of the corresponding diesel fuel, a growing trend in current research has focused on the development of alternative fuels to oil. Among various possible sources, biodiesel is an alternative as a renewable and clean replacement compared to nonrenewable petroleum diesel [1,2]. Biodiesel is constituted by alkyl esters of fatty acids and produced from transesterification reactions of triglycerides and esterification of free fatty acids (FFA), short chain alcohols in the presence of a catalyst to produce alkyl esters (biodiesel) and glycerol [2]. The advantages of biodiesel include its biodegradability, non-toxic nature and a sulfur-free composition. The cost of feedstock is a major economic factor in the viability of biodiesel production [2,3]. Furthermore, if biowaste is used as a feedstock, the production process permits an almost complete recycling of the organic waste [4,5]. The esterification using conventional homogeneous acid catalysts, such as H_2_SO_4_, HCl and HNO_3_ [1,6,7,8,9], provides high conversions into alkyl esters in a short time. 

The development of heterogeneous catalysts and the heterogeneization of homogeneous catalysts have great economic and environmental interest. This is because they may eliminate or reduce the generation of effluents, simplifying the separation of the reactional products and the catalyst, avoiding the use of liquid catalysts that are, many times, toxic to the environment, and are recyclable [9,10,11].

Heteropolyacids (PAHs) with Keggin-type structure have been of great interest in the field of acid catalysis [12], because of its acidic properties and high catalytic activity [13,14]. However, they are unsuitable for esterification because of their high solubility in polar media [15,16,17]. An alternative to overcome this problem could be to support these heteropolyacids on a solid with a high surface area, such as MCM and SBA [9,18,19], metakaolin [9,20], Zirconia [21,22], montmorillonite [13,16,23,24,25,26] and others.

Bronsted acid catalysts and heterogeneous Lewis-acid Montmorillonite are an alternative to homogeneous reactions catalyzed by acid [27,28,29,30]. Montmorillonites are hydrophilic clays of bentonite type, with a layered structure and have been investigated as environmental and reusable catalysts [28,31]. Montmorillonite clay is among various layered aluminosilicates, consists of layers of two tetrahedral silica sheets sandwiching one octahedral alumina sheet, which has caught great attention especially due to its good swelling property and high exchange capacity, which is abundant on the earth [16,32]. The ability of exchangeable ions in their interlayer spaces allows the introduction of various metals, heteropolyacids and physiological functions between the layers, in addition to acid activation [1,13,24,25,33,34,35,36,37]. Both the modified-bentonite and bentonite are promising supports due to their common fascinating features, such as their inherent acidity, excellent thermal stability, and easily controlled structure and morphology [16].

An obstacle to produce biofuels is its high price. Aiming to make the production of biodiesel more interesting, it is necessary to use low cost materials. Biomass residues are unique raw materials that can replace fossil fuels to synthesize both fuels and chemicals [5]. Therefore, it is possible to see the potential of clays and lipid-rich wastes for making useful or potentially useful products as biodiesel. Heterogeneous catalysts based on clays have received considerable attention in different chemical processes due to their environmental compatibility, abundance on the earth, low cost, selectivity, thermal stability and recyclability [32]. 

Modified clays are widely used in acid catalysis [1,7,8,27,28,30,32,38,39,40]. In a previous study in our research group, Nascimento et al. [39] used acid-activated flint kaolin as an effective catalyst for the esterification of distillate of deodorization of palm oil (DDPO) obtaining 92.8% conversion to fatty acid esters (biodiesel). In another paper, Pires et al. [9] impregnated 12-tungstophosphoric (HPW) in a waste kaolin and mesoporous materials (MCM and SBA) obtaining acid catalysts that have been applied to the esterification of DDPO with maximum activity of 83% conversion. Lacerda Junior et al. [20] used 12-tungstophosphoric impregnated in Amazon kaolin flint, resulting in a series of catalysts that were tested in the esterification of oleic acid resulting in 97.21% of conversion. H_3_PW_12_O_40_ and Cs*_x_*H_3−*x*_PW_12_O_40_ have been supported on SiO_2_, MCM-41 and ZrO_2_ that were prepared and applied as solid acid catalysts for the esterification of palm free fatty acids with methanol in a yield of 92% in methyl esters [15]. Dharne and Bokade [24] supported the dodecatungstophosphoric acid (H_3_PO_4_·12WO_3_·*x*H_2_O) in montmorillonite clay type K 10 and applied it as a catalyst to the esterification reaction of levulinic acid with a 97% efficiency of levulinic acid conversion and 100% selectivity to n-butyl levulinate.

12-Molybdophosphoric acid (HPMo) has been widely employed as a solid acid catalyst, being an efficient proton donor and therefore exhibiting excellent catalytic activity with reasonably high thermal stability [41]. Many researchers continue to direct their attention to the development of simple, cheap and reusable catalysts for the synthesis of alkyl esters from low cost waste/biomass feedstocks [5]. As example, Khayoon and Hameed [42] anchored HPMo on SBA-16 to be employed as solid acid catalyst in the esterification of crude karanja oil (CKO), reporting an 82% methyl ester (FAME) conversion for a highly reusable material (four consecutive cycles). Carvalho et al. [43] impregnated HPMo in alumina (Al_2_O_3_). The catalyst obtained was employed in the simultaneous esterification/transesterification of biomass, *Mucor circinelloides* URM 4182, able to convert the microbial lipids into ethyl esters (FAEE) with yields of 97%. 

In another study, Conceição et al. [14] supported HPMo in niobia to produce a solid catalyst employed in a simultaneous esterification/transesterification reaction of macauba oil (*Acrocomia aculeata*) to 99.65% ester, a biodiesel of high quality. The catalyst was reused and maintained an ester content higher than 94% up to the second reaction cycle. HPMo has been indeed shown to be efficient in heterogeneous catalysed processes, mainly in the conversion of triacylglycerol and of free fatty acids into alkyl esters. 

In a review, Timofeeva [17] reported on heteropolyacid catalysts with different structures, which have a potential range of promising applications as acid catalysts in various organic reactions, by having an activity raises more than conventional catalysts known in the literature. The catalytic effects of PAHs in acidic reactions depends on three factors: acidity, heteropolianion structure and the type of reaction. On the other hand, clays may be used as catalyst support [9,13,16,20,25,26,34,41,44], or act as acid catalysts [7,8,38,39], redox catalysts and in removing heavy metals from the environment from modifications [45,46]. Both the modified-bentonite and bentonite are promising supports due to their common fascinating features, such as their inherent acidity, excellent thermal stability, and easily controlled structure and morphology. Therefore, the modified-bentonite with larger specific surface area is widely used as a very good catalyst carrier [5,13,16,24,25,41,47]. Actually, heteropolyacid-impregnated solid acids have caused great interest in many fields.

The present work is mainly focused on reporting the preparation, characterization and the use of 12-Molybdophosphoric acid supported on natural bentonites as efficient catalysts to produce biofuel from a waste industrial residue used as feedstock. HPMo supported on natural bentonites was chosen because the reactions catalysed by that acid proved to be effective in the conversion of triacylglycerol and free fatty acids to alkyl esters [14,42,43]. This is an important topic, since distillate of deodorization of palm oil (DDPO) has a high content of free fatty acids (84%), besides being largely available at a low cost [9,39,48,49]. The esterification of DDPO was selected as a reaction to evaluate, for the first time, the catalytic performance of the catalysts obtained by the method adopted in this work. The influence of various reaction parameters (reaction temperature and time, DDPO-ethanol molar ratio and catalyst concentration) on catalytic performance was studied. The recycling of catalyst was also investigated by reutilization tests.

## 2. Materials and Methods

### 2.1. Raw Material and Chemicals

Two natural bentonite samples from Cubati (BC) and Lagedo (BL) cities located in the state of Paraíba (Northeastern Region of Brazil) were used as starting materials for the preparation of hybrid catalysts. The chemical composition of the samples was obtained by the Dispersive Energy X-ray spectroscopy (EDS) technique (see Table 2). The fraction of clay enriched with montmorillonite less than 2 μm was supported with H_3_PMo_12_O_40_·xH_2_O (designated by HPMo). The reagents were ethanol (EtOH, Nuclear, 99.5%, AR), methanol (Nuclear, AR), 12-phosphomolybdic acid (HPMo, Mo ≥63%, Vetec, Rio de Janeiro, Brazil, AR), hydrochloric acid, AR), sodium bicarbonate (Na_2_CO_3_, Vetec, AR), sodium bicarbonate (NaHCO_3_, Vetec, AR) and sodium hydroxide (NaOH, Vetec, AR). All reagents were used as supplied. The DDPO is a residue (viscosity at 60 °C =12.296 mm^2^ s^−1^; density at 60 °C = 0.862 g mL^−1^; water content <0.5%; oxidative stability >150 h; acidity index =177.15 mg KOH g^−1^) consisting of 84% fatty acids (42% palmitic, 41% oleic, 10% linoleic, 5% Stearic, 1% Lauric and 1% myristic), 12% by weight of triglycerides, diglycerides and monoglycerides and 4% by weight unsaponifiable matter, corresponding to 4% of the product formed in the refining of palm oil [9,39], kindly donated by Companhia Refinadora da Amazônia, Agropalma S/A (Brazil).

### 2.2. Preparation of the Supports and Impregnation with HPMo

The dried and ground natural clays were prepared by successive cycles of separation of the particles in deionized water by ultrasonic disintegration for 5 min, then passed completely in a 0.062 mm sieve. The clay fraction below 2 μm was obtained by gravitational sedimentation of the washed samples. Then, by centrifugation, the precipitate was dried at 65 °C according to the procedures described by [34,50]. 

The catalysts derived from the bentonites impregnated with HPMo were prepared by adopting the procedure reported in the literature [9,13,21,25]. The method involves stirring 8 g of sample in 50 mL of 0.1 mol L^−1^ HCl solution and 2 g of HPMo (25% w/w). The amount of HPMo used was calculated assuming the formation of a monolayer cover on the surface for each support. The mixture was stirred vigorously and heated to 80 °C until complete evaporation of the liquid part. After this time the solid residue was subjected to a temperature of 120 °C for 12 h and then calcined at 200 °C for 4 h at a heating rate of 10 °C min^−1^. The catalysts were named according to their origin of bentonites in BCM (Modified Cubati Bentonite) and BLM (Modified Lagedo Bentonite).

#### Washing of Bentonite Impregnated with HPMo

Heteropolyacids are quite soluble in aqueous medium [17,47]. Thus, esterification reactions using pure HPMo as a catalyst are carried out in a homogeneous phase. The literature also reports that the leaching of heteropolyacids from the support is dependent on the catalyst preparation conditions [9,21]. In order to eliminate the excess HPMo of 25% HPMo (w/w) in the supports (BCM and BLM), which could be responsible for the homogeneous reaction phase, 5 g of sample was used, which was inserted in a Soxhlet extractor with ethanol (500 mL each) under constant stirring for 24 h according to procedure, adapted from [9,51]. At the end of this period the solid was recovered by filtration, washed extensively with water and at room temperature. The obtained solids receive the identifications Modified Cubati Bentonite Washed (BCMW) and Modified Lagedo Bentonite Washed (BLMW). HPMo leaching was then monitored by quantifying the molybdenum in the BCM and BLM supports analyzed by EDS as can be seen in Table 1.

The washing step was important to remove the HPMo units which were not immobilized onto the support during the synthesis. The removal of those Keggin units was necessary to avoid their leaching in the liquid medium during the reaction. In this manner, the measurement of the HPMo in the Soxhlet washing solvent made it also possible to indirectly estimate the amount of HPMo efficiently immobilized [20,21,51]. By spectroscopic analysis of ultraviolet-visible spectroscopy (UV-vis) the quantities of HPMo were estimated in the recovered ethanol after extraction and thus, by difference, the quantities of HPMo that were effectively incorporated in the matrices BC and BL [20,21,51]. 

### 2.3. Characterization

Chemical analysis of the samples was obtained by EDS microanalysis using an apparatus trade mark Oxford, model Aztec Energy X-Act (Brno, Czech Republic). The loss on ignition analysis was made by gravimetry. 

X-ray diffraction (XRD) patterns were obtained on a diffractometer PANalytical (Almelo, The Netherlands) X’PERT PRO MPD (PW 3040/60) with CuKα radiation at 40 kV and 30 mA. The scan was made 0.02°/10 s and the 2θ values were analyzed in the region of 3° to 35°. 

N_2_ adsorption–desorption isotherms were obtained at liquid nitrogen temperature using a Micromeritics TriStar II model 3020 V1.03 apparatus (Norcross, GA, USA). Before each measurement, the samples were outgassed at 130 °C for 2 h. The specific surface area, the microporous area, the microporous volume and the pore-size distribution were obtained, respectively, using Brunauer-Emmett-Teller (BET), t-plot and Barrett-Joyner-Halenda (BJH) methods. 

Fourier transform infrared spectroscopy (FTIR) spectra were obtained from a spectrophotometer of Shimadzu (Kyoto, Japan) model IRPrestige-21A with a resolution of 32 and 100 scans and analyzed by Thermo Electron Corporation, IR 100 model with a resolution of 4 and 32 scans. For the analysis of all materials KBr pellets were used and the spectra were obtained in the region 4000–400 cm^−1^. 

The thermogravimetric analysis (TGA/DTG) curves were obtained on a Shimadzu DTG-60H model using N_2_ as purging gas (50 mL min^−1^). The analyses were performed from room temperature (~25 °C) to 1000 °C at a rate of 10 °C min^−1^.

Morphological analysis of the samples was obtained by an apparatus of Tescan, VEGA 3 SBU model (Brno, Czech Republic) operating at 20 kV and 7.5 mA. The samples were supported on carbon tapes and metalized with gold under vacuum conditions. 

FTIR of adsorbed pyridine was the technique used to confirm the presence of Brønsted and Lewis acid centers in the catalysts [27,52,53]. A Shimadzu model IRPrestige-21A was used. 

**Figure 1:**
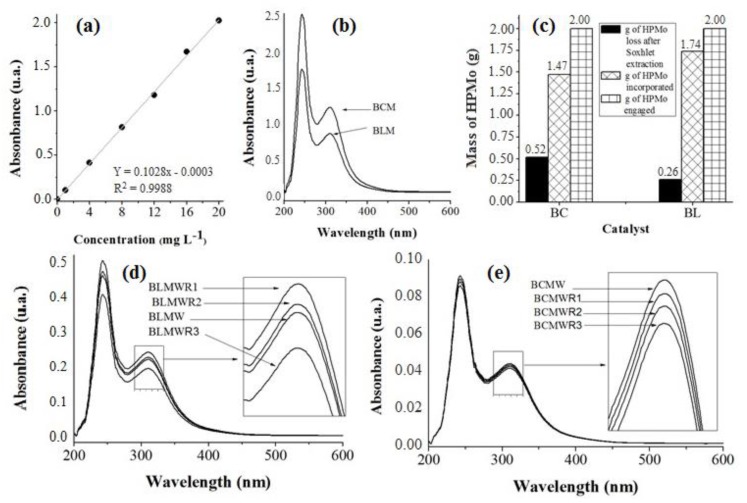
(**a**) Calibration curve for determination of the leaching of the catalysts supported with heteropoly phosphomolybdic H_3_PMo_12_O_40_ (HPMo); (**b**) ultraviolet-visible spectroscopy (UV-vis) spectra of the solution after washing with ethanol for 24 h; (**c**) quantification of HPMo losses by UV-vis spectroscopy and calculation of immobilized HPMo; (**d** and **e**) UV-vis spectra of the solution after reaction.

Tests to determine the leaching of the HPMo of the catalysts were performed in a UV-vis equipment of Thermo-scientific (Waltham, MA, USA), model Evolution array UV-vis spectrophotometer, with a scan of 200 to 800 nm and resolution of 1 nm. The liquids were placed in a quartz tube. The calibration curves were constructed at the wavelength 310 nm (λ_max_) [54], analyzing the solution of HPMo in ethanol at known concentrations (Figure 1a). Based on literature [20,21,51], the masses of HPMo immobilized in the supports were determined. 

Acidity determination was performed using acid-base titration. For this purpose, 0.10 g of solids were dispersed in 20 mL of 0.1 mol L^−1^ NaOH under agitation for 4 h. Acidity was determined by titration with a 0.10 mol L^−1^ HCl solution [14,55]. The content of Brønsted acid sites for the catalysts used was determined by the modified Boehm titration method [56,57], which quantify the oxygenated functional groups that are adsorbed on the surface of the catalysts. 0.10 g of the catalysts were dispersed in 20 mL of each solution: NaOH (0.1 mol L^−1^), which reacts with carboxylic, lactonic, phenolic and Brønsted acid (HPMo) groups; Na_2_CO_3_ (0.05 mol L^−1^), which reacts with reacts with the carboxylic and lactonic groups; and NaHCO_3_ (0.05 mol L^−1^), which reacts only with carboxylic groups [56,57,58].

### 2.4. Reaction Procedure

Prior to the experiments, the catalysts were activated at 130 °C for 2 h. Tests of the catalysts were conducted in one run on a multirreator PARR 4871 (Parr Instrument Company, Moline, IL, USA). In a typical experiment, the DDPO was mixed with alcohol at the molar ratio of 1:30 (DDPO:alcohol) and 5% w/w of the solid acid catalyst (As compared to the mass of DDPO). The reaction mixture was stirred (500 rpm) and warmed from room temperature to 160 °C. Once the desired temperature was reached, the system was maintained for 120 min. This time was considered as the kinetic contact time. At the end of the reactions, the catalyst was filtered off. The percent conversion of DDPO to the corresponding ester was estimated by an acid measure of the product by titration with 0.1 mol L^−1^ hydroxide according to the method described in the literature [9,39,49].

### 2.5. Leaching Tests

The leaching tests were performed as described elsewhere [23,42] with some modifications. The catalysts were treated with hot ethanol (160 °C, 2 h, in multirreator). The ratio of catalyst:alcohol was the same as in the catalytic tests. The hot filtrated ethanol was then used for the esterification reaction as a reactant under the conditions described above but without any catalyst. 

## 3. Results

### 3.1. Chemical Composition of Samples by EDS and Heteropolyacids Immobilization inside the Matrix

The chemical composition data presented in Table 1 showed the quantification of the elements present in the samples, and showed that they have values similar to those obtained by previous studies for compounds of montmorillonite type [36]. The concentration of Mo in the BCM (Modified Cubati Bentonite) and BLM (Modified Lagedo Bentonite) samples was 18.15% and 17.72%, respectively. Although the nominal percentage of Mo initially was 16%, it was observed that the actual percentages for modified samples were slightly above this theoretical value. As the method of analysis by EDS (spectra in the Figure S1 in Supplementary Material) is performed in scanning the samples, some points were possibly measured with a higher concentration of Mo. After washing with ethanol, the percentages of major elements in the BCMW and BLMW samples changed significantly compared to the observed values before washing [9,51]. Although the washing process was carried out under the same conditions for the two samples, the variation in the chemical composition showed that the washing was more effective in the BCMW sample, which had a reduction of 18.15% to 15.01% in relation to the Mo, while in BLMW the reduction was insignificant. This difference can be related to the interaction of HPMo with the supports and their surface areas.

The BL sample exhibited the largest surface area (43 m^2^g^−1^) while BC presented a reduced surface area (26 m^2^g^−1^). A smaller area may have facilitated the removal of Mo by ethanol during the washing of modified BCM and BLM supports [59]. The presence of molybdenum in the washed samples (BCMW and BLMW) confirms that the impregnation technique was successful, especially for BLM, where the heteropolyacid is present in the layers of this support and possibly within the pores [9,13] as observed in an evident derterioration of its textural properties in Table 2. 

### 3.2. Heteropolyacids Immobilization on BC and BL

**Scheme 1:**
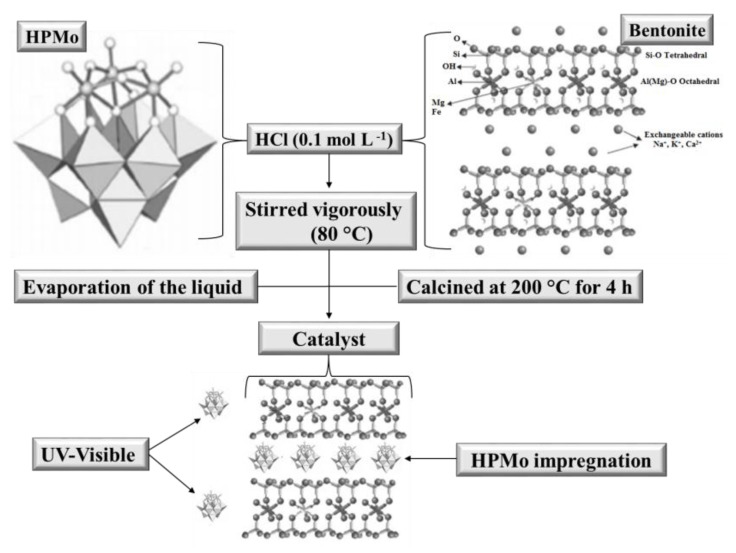
Bentonite modified with phosphomolybdic heteropolyacid catalyst synthesis procedure.

UV spectroscopy analysis of the supernatant (Scheme 1) from the washing of modified samples (BCM and BLM) provided an estimation of the quantities of HPMo not incorporated in the solids (Figure 1a). Incorporated HPMo quantities into the matrices could be calculated by subtracting the initial amount of HPMo added minus the amount of HPMo detected by UV-vis in the supernatant [51].

UV-vis spectra of modified BCM and BLM samples shown in Figure 1b revealed absorption bands at 245 and 310 nm, attributed to O-M load transfer range of the molybdophosphate anion. This indicates the presence of non-degraded Keggin phases referring to heteropolianion [PMo_12_O_40_]^3−^ (Figure 1b,d,e), as already reported in literature [51,54]. According to data obtained from Figure 1c, BLMW and BCMW exhibited total HPMo losses of 13% and 26%, respectively. The mass percentages of HPMo supported in solid catalysts were recalculated as 74% (1.47 g) for BCMW and 87% (1.74 g) for BLMW. No appreciable decrease was observed in HPMo content by analyzing the UV-vis spectra of the catalyst samples employed after three cycles of DDPO esterification (Figure 1d,e). BLMWR (R = Reused) samples showed lower HPMo losses as compared to BCMWR, which evidenced a better interaction and stability of HPMo on BLMWR.

### 3.3. XRD Analysis

**Figure 2:**
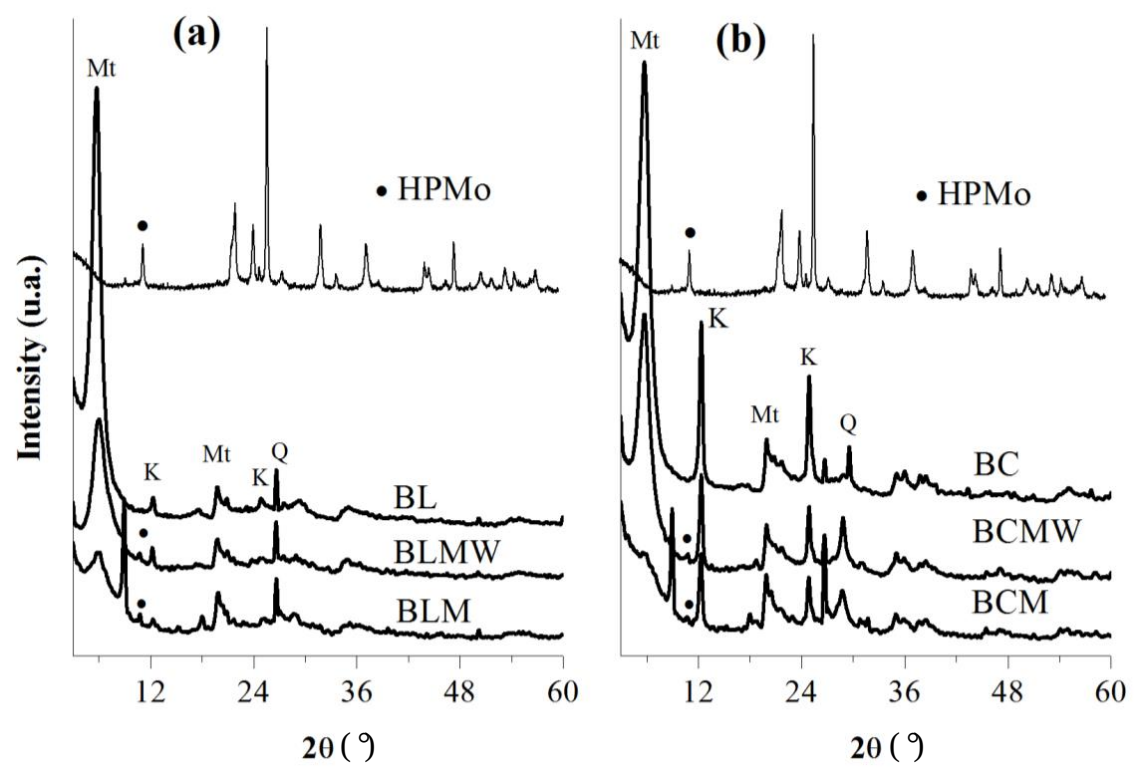
X-ray diffractograms of the samples (**a**) Lagedo Bentonite (BL), Modified Lagedo Bentonite (BLM), Modified Lagedo Bentonite Washed (BLMW) (**b**) Cubati Bentonite (BC), Modified Cubati Bentonite (BCM), Modified Cubati Bentonite Washed (BCMW). The X-ray diffraction (XRD) pattern of HPMo.

XRD patterns of natural bentonites (Bent) (Figure 2a,b) showed peaks related to montmorillonite (Mt), Kaolinite (K) and quartz (Q) cristals, an importante fingerprint for compounds that belong to the smectite group [34,44,50,60].

BL and BC samples exhibited high levels of argilomineral Mt. In addition to the peaks at 2θ = 5.93° and 19.9° (14.90 and 4.44 Å) from Mt, other additional well defined and intense peaks are observed mainly in BC (Figure 1b), with reflections at 2θ = 12.35° and 24.90° (7.09 and 3.57 Å) and 2θ = 27° (3.32 Å) corresponding to kaolinite and quartz, in that order [7,8,38], similar to those observed in South American bentonites [60,61]. The kaolinite phase was observed in BL with less intense reflections, possibly due to the high degree of structural disorder and small crystal size [62]. This may have contributed to the higher porosity and increase in BL surface area (Table 2).

In the case of BCM and BLM (Figure 2a,b), HPMo impregnation originated an intensity reduction of the main peaks, with a distance at d001 around 16.73 Å (2θ = 5.28°). This d_001_ value is slightly higher as compared to that previously obtained for BC and BL (d001 = 14.90 Å) and can be attributed to the presence of HPMo (2θ = 9.02°) in the interlamellar space and inside the pores of the supports [13,25,29,45] that possibly caused changes in morphology [29] (Figure 5). XRD patterns of impregnated and washed bentonite are also shown in Figure 2. After washing the samples (BCM and BLM) to remove the excess of non-impregnated HPMo, the montmorillonite peaks of the washed samples (BCMW and BLMW) showed a d001 (15.38 Å) interplanar distance comparably higher to that of the parent materials (BC and BL).

The crystallographic pattern of kaolinite is also present in all the diffractograms of Figure 2a,b. Although BCM and BLM samples were calcined at 200 °C, this temperature is not sufficient to convert kaolinite to metakaolinite [62,63]. The metakaolinite phase is evidenced by the presence of an amorphous SiO_2_ phase in a 2θ = 10°–20° range and by the absence of the kaolinite peak [7,8,38,63], which was not observed in any of the diffractograms.

There was no goethite, illite or other phases in the studied samples that could be sources of iron oxides and hydroxides. Therefore, only reflections corresponding to Mt, K and Q were observed. Therefore, iron present in the crystalline structure of Mt in bentonites samples may possibly be associated with silt (fine quartz particles) and kaolinite particles or agglomerates of Mt particles [60]. Another possibility is due to the Fe–OH bond generated after isomorphic substitutions of octahedral aluminum atoms by iron atoms in the structure of kaolinite [62], mainly in BL, BLM and BLMW samples that presented very high iron contents in comparison to samples BC, BCM and BCMW according to EDS analysis (Table 1).

After modification of the natural bentonites as described in Section 2.2, BLM and BCM materials were calcined at 200 °C. The high intensity reflections of crystalline HPMo at 2θ ≈ 10° can be observed in the BLM, BCM, BLMW and BCMW XRD standards. This implies that the HPMo is not well dispersed on the surface of BCM, BLM, BCMW and BLMW, and probably may be in the form of crystallites containing water molecules, i.e., H_3_PMo_12_O_40_ and H_3_PMo_12_O_40_·13H_2_O [13]. In the diffractograms with HPMo loading for BL and BC, the peaks were observed to be less intense, indicating that BLMW and BCMW have less crystallinity with respect to BL and BC upon HPMo impregnation [13,25]. The comparison between the diffractograms of solids BC, BL, BCMW and BLMW (Figure 1) are very similar, revealing that the structure of bentonite is maintained even after washing. The HPMo peak is more intense in the BCM sample, which characterizes the presence of agglomerates on the surface of that sample, which can be observed in Figure 5, while in the larger surface area BLM sample, the HPMo crystals may be more dispersed between the pores of this material [16,22], which could justify the presence of less intense peak for HPMo in this sample.

### 3.4. Thermogravimetric Analysis (TGA/DTG)

**Figure 3:**
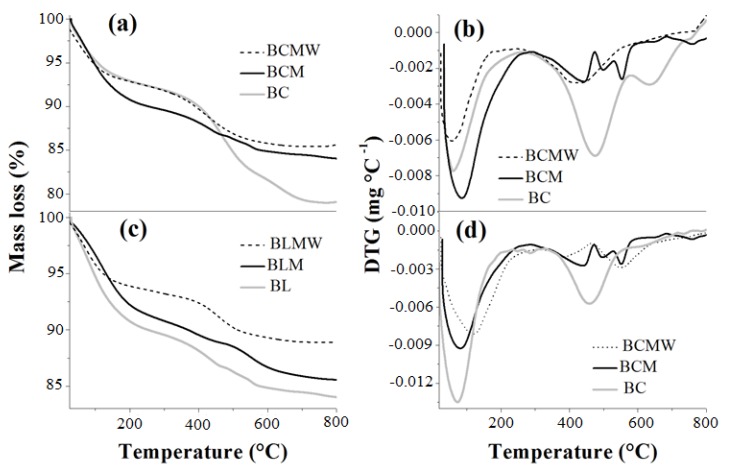
Thermogravimetric analyses TGA and DTG curves; (**a**,**b**) for BC, BCM and BCW samples; (**c**,**d**) for BL, BLM and BLMW samples.

The thermal stability of the supported catalysts was analyzed by thermogravimetric analyses (TGA/DTG). Pure BC and BL samples presented a similar profile to that of decomposition of the supported samples BCM, BCMW, BLM and BLMW as can be seen in Figure 3a–d. The derivative curves (Figure 3b–d) provided information on the different steps of the decomposition of the natural and modified clays. At temperatures below to 150 °C there was a weight loss attributed to desorption of water adsorbed on the external surface or coordinated with interchangeable cations between the clay mineral layers [34,41,44,50]. That weight loss can also be attributed to the inclusion of hydrated HPMo in the pores of the supports [41,59]. The possible presence of water is confirmed by FTIR spectra bands at 3443 and 1630 cm^−1^ (Figure 5b) that were attributed to stretching vibration of the OH group and angular deformation of HOH which are, probably, adsorbed waste water in the interlayer spaces [34,50]. 

In the range of 200 to 500 °C, the observed weight losses may indicate the decomposition of organic matter [64], the loss of water molecules that were chemically absorbed between the layers of the support, and the strong interaction of HPMo with species of Mg^2+^ and Ca^2+^ present in the media, resulting in crystallization of MoO_3_ close to 430 °C [59]. The mass loss from 500 to 800 °C could be related to the dehydroxylation of the chemically bound water. Temperatures in the range of 530 to 565 °C are probably associated with the dehydroxylation of the argilominerals, Mt and k present in the samples [60,62]. Since the argilominerals in question were identified by X-ray diffraction, (Figure 2a,b), and the samples BC, BCM and BCMW showed very strong peaks related to kaolinite with higher mass loss, except for the BCMW sample. It can be associated to the presence of iron and aluminum ions in the clay mineral. 

Analyzing DTA curves, all observed events were endothermic and they can be attributed to loss of physisorbed water and adsorbed gases (<200 °C) as well as structural dehydroxylation (>200 °C) of kaolinite present in these samples (detected by XRD) [37,50,60,64]. Generally, TGA/DTG profiles for the hybrid catalysts were similar to the corresponding supports (bentonites). Thermal effects associated with the decomposition of HPMo are analogous to the degradation steps of Bentonite [13]. This may be due to the formation of intermolecular binding between the support and the heteropolyacid and indicated the presence of chemical interactions between them, a behavior similar to that previously reported for HPMo deposited in montmorillonite [13,41].

Chemical analysis (Table 1) and FTIR vibration spectra peaks near to 1084, 785 and 690 cm^−1^ (Figure 5) confirmed that these silanol groups (Si–OH–Al, Si–OH–Fe) exists in the samples, even after the incorporation of HPMo [13,33,35,41]. The presence of the HPMo in the prepared materials was also confirmed by FTIR as can be seen in Figure 5. Keggin-type structure characteristic bands appeared at around 1040, 965, 866 and 780 cm^−1^ and are characteristic of heteropolianions (PMo_12_O_40_)^−3^ [13,14,41,65]. Moreover, chemical analysis data (Table 1) reveals the existence of species Mo and P in the samples.

### 3.5. Textural Properties

**Figure 4:**
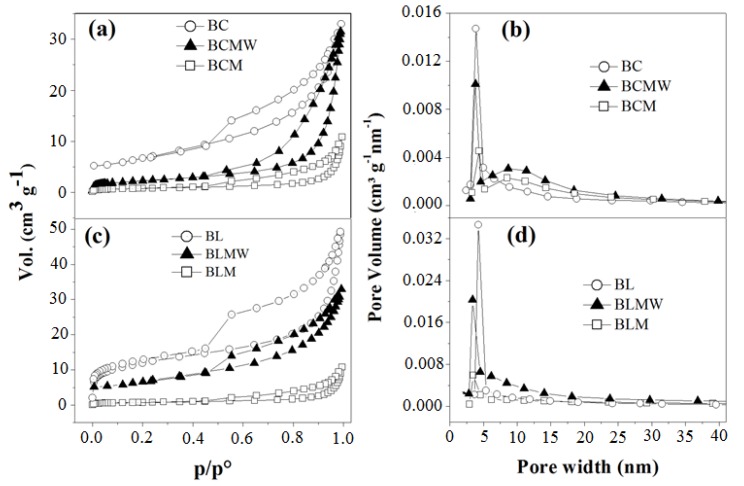
Distribution of the pore size and the N_2_ adsorption-desorption curve of the natural, modified and washed samples. (**a**) BC, BCM, BCMW; (**b**) BC, BCM, BCMW; (**c**) BL, BLM, BLMW; (**d**) BL, BLM, BLMW.

Most isotherms of Bentonite samples (Figure 4a,c, with the exception of BCM and BLM) were of type IV according to the IUPAC classification. The separation of the adsorption/desorption curves in the relative pressure (close to 0.4) indicates the presence of small mesopores in the adsorbate [45]. The adsorption-desorption analysis of N_2_ indicates the formation of mesoporous materials with the addition of 25% of HPMo in the supports [13,45]. The pore size distribution curves obtained by the BJH method [13,25,45] for the samples BC, BL, BCM, BLM, BCMW and BLMW are shown in Figure 4b,d, indicating that there is a narrow range of pore distribution (4 nm) for samples BC and BL corresponding to mesoporous materials [45]. With acid treatment (HCl) there is a very clear distribution of pore size in the 2–25 nm range for samples BCMW and BLMW, respectively (Figure 4b–d), probably due to leaching of aluminum and magnesium, as can be observed in Table 2, causing a decrease in Mt reflections of BCM and BLM materials [27,28], as observed by the XRD technique (Figure 1a,b). Importantly, BCM and BLM exhibited a typical non-porous profile with only certain interparticle macroporosity as shown in Figure 4 and Table 2.

The presence of a hysteresis demonstrated the strong interaction between the surface and the fluid in a mesoporous material (2–50 nm) formed by several layers [59]. BC and BL samples showed similar isotherms, and their surface areas were 24 and 43 m^2^ g^−1^, respectively. Comparing the curves of the samples, BL adsorb/desorb a larger volume of N_2_, due to the higher surface area presented by this material, as shown in Table 2. The BC sample had a low surface area in relation to BL due to its high content of kaolinite (Figure 2). It is observed that the addition of HPMo to BC and BL causes a small reduction in the volume of nitrogen adsorbed over the range of P/P° values, similar to those observed for bentonite (Campina Grande, Paraíba State, Northeastern Brazil) [45]. This effect may be related to the deposition of HPMo on the surface of BCM and BLM that probably blocked the access to some pores, causing an almost complete collapse of the structure, as observed by electron micrographs (Figure 6), rendering essentially non-porous materials. Similar observations were reported by other authors, who employed several materials besides clay-minerals as substrates for heteropolyacids [9,13,14,16,20,21,25,41,42,47].

### 3.6. FTIR Spectroscopy Analysis

**Figure 5:**
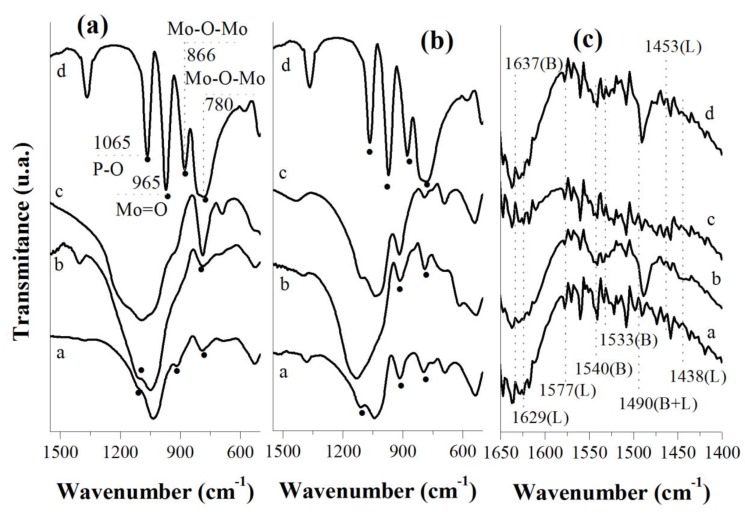
Fourier transform infrared spectroscopy (FTIR) spectra for samples: (**a**) a—BLM, b—BLMW, c—BL, d—HPMo; (**b**) a—BCM, b—BCMW, c—BC, d—HPMo; (**c**) samples treated with pyridine, a—BL, b—BLMW, c—BC, d—BCMW.

In order to determine the presence of HPMo materials incorporated into the materials, FTIR spectroscopy analysis was performed. The spectra of HPMo, BL, BLMW, BC and BCMW are shown in Figure 5a,b. FTIR analysis showed peaks in the spectral region of 520, 675, 787, 917, 1030 and 1093 cm^−1^ which are bending vibrations of symmetric and asymmetric TOT (tetrahedral layers of silicon coordinated with oxygen) (T = Al, Mg, Si and Fe) opening and inside the pores [28]. The peak at 787 cm^−1^ is due to Si–O–Si vibrations and FTIR spectrum in the region of 917 and 675 cm^−1^ are derived from OH groups bound to silanol groups (Si–OH–Al) [35] and influenced by the presence of Fe ions [33], which remain after the acid impregnation process, they are elements which are part of the bentonite structure. The characteristic bands of Keggin structure (HPMo) appearing at around 1065, 965, 866 and 780 cm^−1^ were the same for BLMW and BCMW samples [13,14,41,42,54,65]. The differences in the intensities are minimum and can be explained by the more efficient impregnation of the heteropolyacid in the BLMW and BCMW samples confirmed by the presence of shoulders at around 1040, 918 and 534 cm^−1^, indicating the formation of MoO_3_ during the impregnation processes [13]. The similarity between the spectra of BLM and BLMW confirms the presence of crystals in both catalysts also confirmed by XRD (Figure 2). The vibrations are clearly observed in BLMW and BCMW spectra. This strongly indicates that the primary structure of the HPMo Keggin anion is preserved even after anchoring to BL and BC supports and calcination at 200 °C. 

### 3.7. Acidity of the Modified Materials

FTIR analysis of pyridine adsorbed allowed the identification of the nature of the sites (Brønsted (B) and Lewis (L)) on the surface of the catalysts BLMWPy and BCMWPy (Figure 5c). Bands attributed to pyridine coordinated to Lewis (L) sites could be observed at 1438, 1453, 1577 and 1629 cm^−1^ in both BLMWPy and BCMWPy materials. The presence of Brønsted sites was also evidenced in absorption bands around 1533, 1540 and 1637 cm^−1^. The band at 1489 cm^−1^ is characteristic of the pyridine ion and M-OH-M-type sites to which pyridine could simultaneously bind to H (B site) and metal atoms (L site) [27,51,52,53]. 

The values found for the surface acidity of the catalysts are presented in Table 2. The amount of cations present in the samples was correlated with the consumption of hydroxyl groups during the titration [8,9]. Catalysts from clay minerals have a heterogeneous distribution of active sites on its surface [8]. With the modification of BC and BL samples, a better distribution of cations from the HPMo in the pores was expected. The addition of HPMo to BL and BC samples increases the number of acidic sites (Table 2, acicity values) as expected and previously reported in the literature for several heteropoliacids deposited in montmorillonites [13,16,24,25,41,47]. BLMW exhibited a larger number of active sites (12.5 mmol H^+^ g^−1^) confirmed by the conversion of 93.3% to 95.5% into ethyl and methyl esters, respectively, in agreement with surface acidity results (Table 3). 

The purification procedure used to remove excess heteropolyacid showed no significant changes in conversion for modified (BCM and BLM) and washed (BCMW and BLMW) samples, according to results from Table 2. 

A comparative study was additionally carried out to study the effect of the supports on the conversion of DDPO. From the obtained results, BLMW exhibited an improved catalytic performance as compared to BCMW in terms of conversion. The effect of the support can be correlated with the acidity data presented in Table 2. BL solids (5.0 mmol H^+^ g^−1^) and BC (4.2 mmol H^+^ g^−1^) have very close surface acidity. BCMW solids (9.2 mmol H^+^ g^−1^) and BLMW (12.2 mmol H^+^ g^−1^) exhibited distinctive surface acidity. The values of the obtained Turnover Frequency (TOF) for these solids followed the same surface acidity trend. Since esterification requires catalysts with acidic sites [9,13,14,41,42,43], the results confirm that the acidity of the supports was a critical factor to maximize the catalytic activtity of the materials in the conversion of DDPO [9,39,49].

### 3.8. Scanning Electron Microscopy (SEM) Results

**Figure 6:**
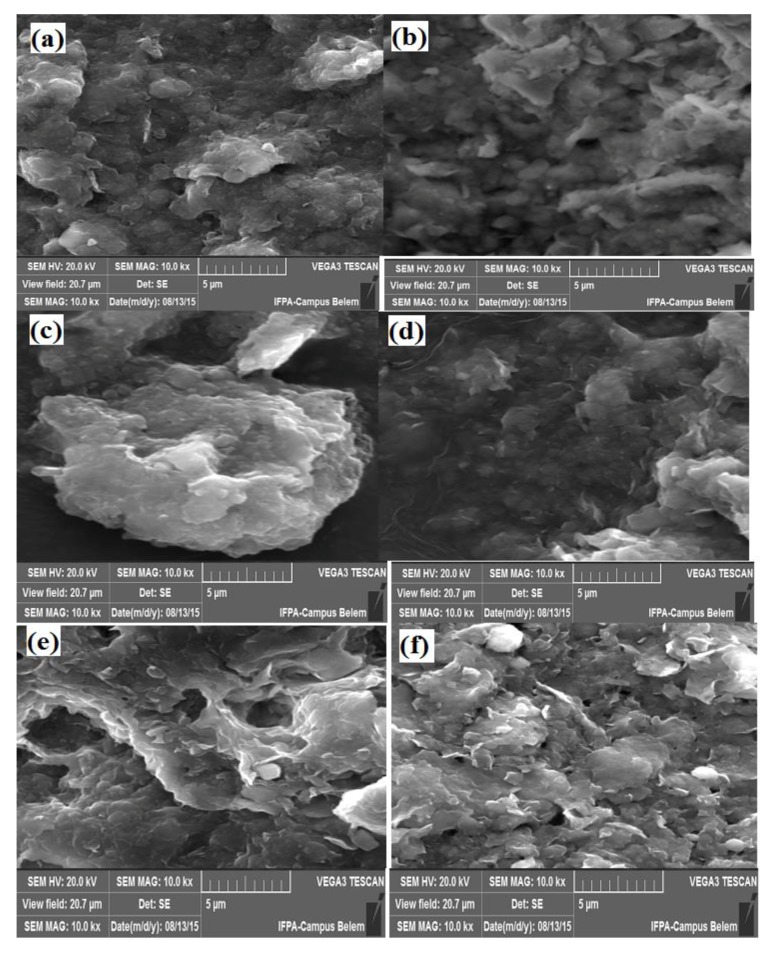
Scanning Electron Microscopy (SEM) micrographs for samples: (**a**) BC, (**b**) BCM, (**c**) BCMW, (**d**) BL, (**e**) BLM (**f**) BLMW.

Scanning Electron Microscopy (SEM) micrographs presented in Figure 6 showed that the bentonites are constituted of particles of irregular plate-like agglomerates as observed in other works [13,25,37,64]. However, for the samples treated with the HCl and HPMo solution and washed (with ethanol), this process did not significantly affect the morphology of montmorillonite particles as shown in Figure 6b,c,e,f. This means that a decrease in specific surface area of montmorillonites after HPMo deposition is predominately associated with changes in their internal pore structure [13], which is not reflected in SEM images. There is a greater agglomeration of the particles and greater irregularity in the dispersion of sizes after the modifications. This is due to the aggregation of the crystals deposited on the outer surface of BCM and BLM due to the interactions of Mt with HPMo [15,59]. Even with the washing of BCM and BLM samples, which showed a certain percentage of leached HPMo, no difference in the morphology of the BCMW and BLMW catalysts was observed (Figure 6c,f).

### 3.9. Catalytic Activity Results

The esterification of DDPO with ethanol or methanol is an electrophilic substitution reaction. It is a relatively slow reaction and needs activation either by higher temperature or by a catalyst to achieve higher conversion [47]. Previous experiments [9,39,49] and preliminary experiments led us to use the considered optimum catalytic conditions, i.e., 25% HPMo, BLMW and BCMW to conduct the proposed esterifications (Table 3).

All materials exhibited high catalytic activity and the results of catalytic tests of the prepared materials for esterification of DDPO are presented in Table 3. The conversion values for reactions without catalyst were 15.5% and 18.6%, using ethanol and methanol, respectively. The BLMW sample exhibited higher activity with respect to BCMW. This result can be explained due to its larger number of active acid sites (9.52 versus 12.6 mmol H^+^ g^−1^). BL stood out as an excellent catalyst support to HPMo and its higher catalytic activity was expected from the results of characterization analysis. Other relevant information relates to Mo content in the samples. Based on the obtained results, an impregnation of 15% (w/w) of HPMo could be enough to obtain optimum catalytic activity. Moreover, the washing was not effective to leach Mo or decrease the conversion. The impregnation of HPMo on BL was indeed very successful, with a significant potential for further studies.

BL and BC samples impregnated with HPMo appeared to be very active catalysts for the liquid phase esterification of DDPO with ethanol or methanol. Additionally, comparative results for the esterification of fatty acids with ethanol or methanol on typical solid acid catalysts reported in literature have been included in Table 4.

BL and BC supports, without the addition of HPMo, exhibited a low catalytic activity. HPMo modified bentonites reached 88.3% (ethanol) and 94.3% conversion (methanol) in the esterification of DDPO. Some other factors such as reaction temperature and time, molar ratio of acid to alcohol and catalyst amount also influenced the conversion in the systems. The variation of different reaction parameters was studied for BLMW and BCMW catalysts in the esterification of DDPO with ethanol, selected as a renewable, abundant and greener solvent compared to methanol and other alcohols [9].

#### 3.9.1. Influence of Reaction Temperature

**Figure 7:**
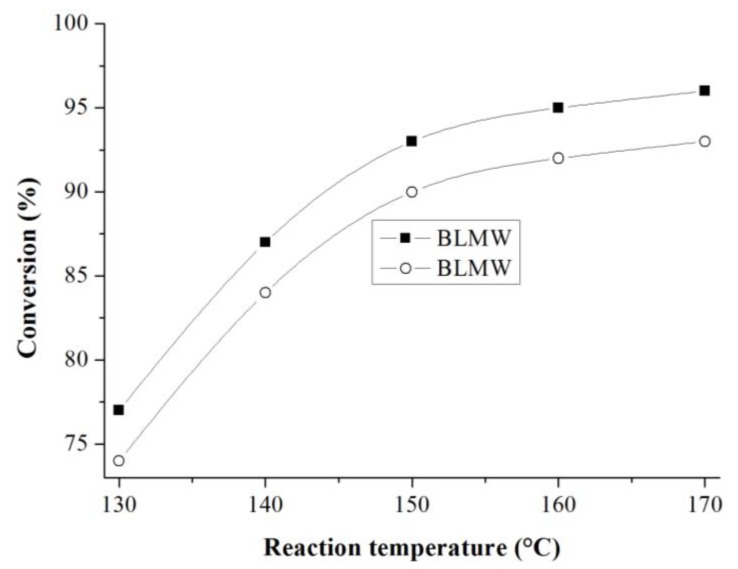
Effect of reaction temperature (°C) on the % conversion during DDPO esterification. Reaction conditions: molar ratio of DDPO:EtOH: 1:30, catalyst amount: 5.0%, and reaction time: 2 h.

The effect of temperature on DDPO conversion was studied. As expected, an increase in temperature from 130 to 170 °C improved the conversion in the systems (Figure 7) [6,7,8,9]. At higher temperatures, conversions remained almost unchanged. DDPO conversion reached 93.3% for BLMW and was 90.7% for BCMW at 160 °C, chosen as optimum temperature for further studies. Optimized conditions for the esterification of DDPO for BLMW and BCMW were: DDPO: ethanol molar ratio of 1:30; 5% of catalyst; 160 °C and 2 h.

#### 3.9.2. Influence of Catalyst Amount

**Figure 8:**
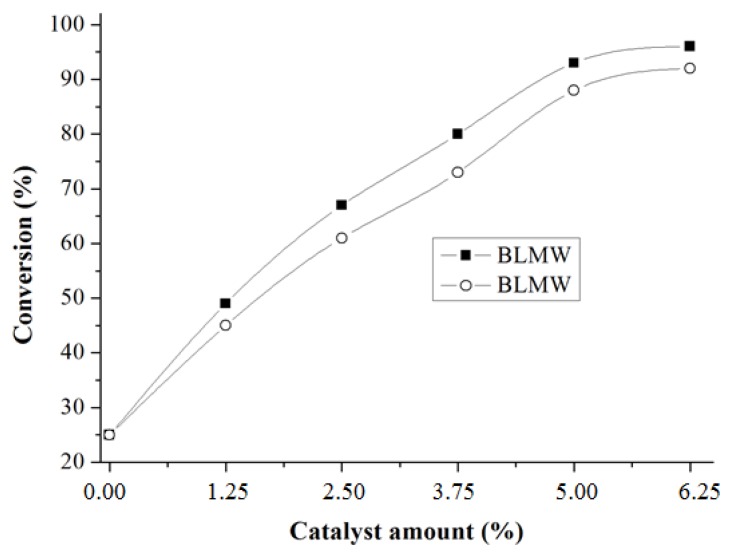
Effect of catalyst amount % fed to the reactor on the % conversion during DDPO esterification with ethanol using catalyst. Reaction conditions: reaction temperature: 160 °C, molar ratio of DDPO:EtOH: 1:30, and reaction time: 2 h.

The effect of the catalyst quantity was investigated by varying the catalyst amounts from 1.25% to 6.25% in relation to DDPO mass (Figure 8). Results indicated that 5% of catalyst provides active acidic sites in enough quantities for the esterification of DDPO. Catalytic reactions on montmorillonites usually involve adsorption and diffusion of reagents through the pores and interlayers of the solid. Reagents diffusion to the active sites can become a limiting process in such solids porous acids when the amount of catalyst exceeds 5% [41,42].

#### 3.9.3. Effect of Molar Ratio

**Figure 9:**
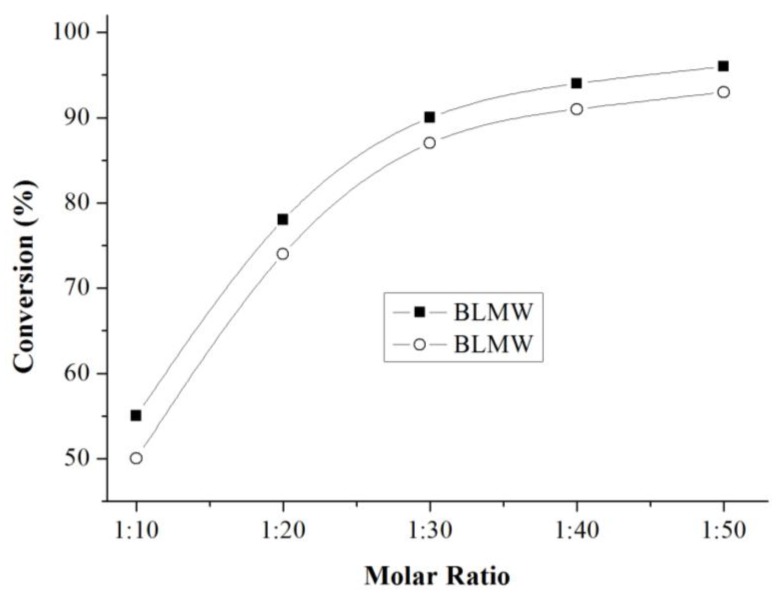
Effect of DDPO:EtOH molar ratio on the % conversion during DDPO esterification with ethanol using catalyst. Reaction conditions: reaction temperature: 160 °C, catalyst amount: 5.0%, and reaction time: 2 h.

To test the effect of DDPO:EtOH molar ratio, the esterification was performed varying the molar ratio from 1:10 to 1:50 (Figure 9). DDPO conversion increased with an increase in the DDPO:EtOH molar ratio, to reach maximum values of 88.9% and 92.5% for BCMW and BLMW, respectively (1:30 molar ratio). With an additional increase in molar ratio, there is only a small increase in conversion, possibly due to the hydrolysis of some of the FAEE produced in the presence of excess ethanol [9,42]. The molar ratio of 1:30 was chosen for further studies.

#### 3.9.4. Effect of Reaction Time

**Figure 10:**
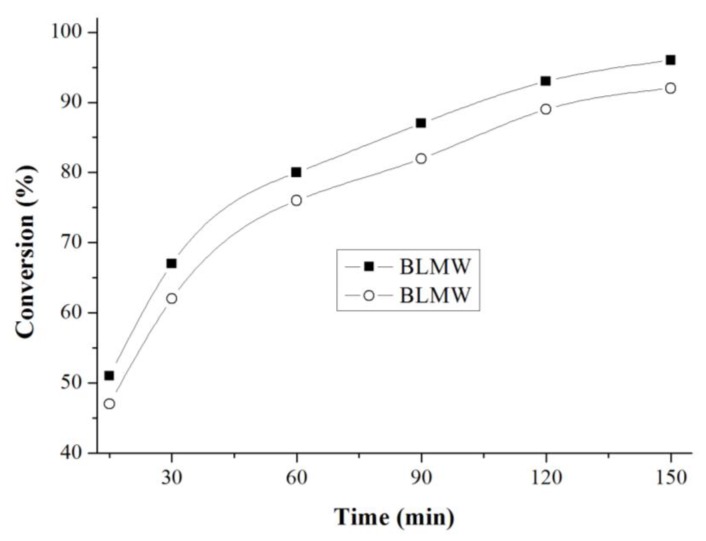
Effect of reaction duration (min) on the % conversion during DDPO esterification over catalyst. Reaction conditions: reaction temperature: 160 °C, molar ratio of DDPO:EtOH: 1:30, and catalyst amount: 5.0%.

The effect of reaction time on DDPO conversion was subsequently studied and results are presented in Figure 10. The conversion of DDPO increased with reaction time. After 15 min of reaction, conversions are relatively low (i.e., 53–60%). Maximum DDPO conversions (93.3% and 90.7% for BLMW and BCMW, respectively) could only be achieved after 6 h reaction. DDPO conversion is almost quantitative within 2 h of reaction for both catalysts. With the additional increase in reaction time there is no significant increase in DDPO conversion. Comparatively, these results significantly improved as compared to results from, Wimonrat [15], Khayoon [42], Wan, Lim and Hameed [68] and Wan and Hameed [69], for which similar conversions could only be achieved after 3–5 h reaction employing similar supported heteropolyacid catalysts in esterification reactions.

Comparing DDPO conversion results in this work to those previously reported (Table 4), certain advantages of the present bentonite systems stand out. Despite high conversion values reported in literature, namely 62% [66] and 99.9% [67] under mild temperatures (50 and 60 °C) and 90% [48] at 130 °C (with the molar ratio and reaction time minimized), these works employed sulfuric acid as catalyst, with its known disadvantages including corrosivity, toxicity and difficulties to recover.

12-tungstophosphoric acid supported on metakaolin (25% HPW/MK700) [9] exhibited considerably high conversions at a high temperature (200 °C) despite the minor molar ratio of reactants. Comparatively, CrWO_2_ and CrWTiO_2_ catalysts required a temperature of 170 °C and a reaction time of 3 h to obtain high conversions (>80%), even though a small molar ratio of reagents is used [68,69], while a catalyst made of the kaolin residue functionalized with –SO_3_H groups) achieved excellent catalytic efficiency (98% conversion) [49] under similar reaction conditions to those employed in this work. Therefore, the present catalysts showed high DDPO conversions under comparatively more benign reaction conditions to most catalysts listed in Table 4.

#### 3.9.5. Reaction Kinetics

**Figure 11:**
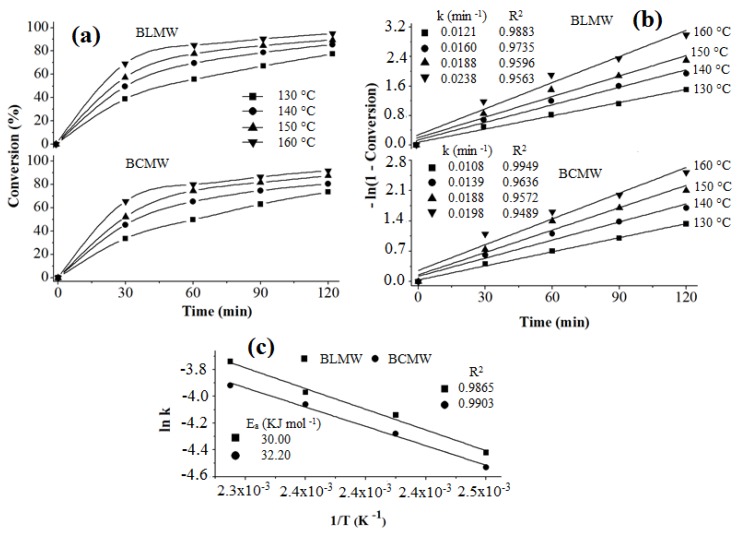
(**a**) The effect of time on conversion using the reaction conditions: Temperature 130–160 °C, DDPO:EtOH, 1:30 (molar ratio), catalyst amount 5.0%; (**b**) Plots of –ln (1 − conversion) versus time at different temperatures; (**c**) Arrhenius plot of ln(k) versus 1/T.

A kinetic study was carried out for the esterification of DDPO on the BLMW and BCMW solids. The reactions were conducted to a molar ratio DDPO:EtOH of 1:30, with 5% catalyst, at temperatures of 130, 140, 150 and 160 °C, in 30, 60, 90 and 120 minutes (Figure 11a). At 120 minutes of reaction, the conversion of DDPO reached 73.5% (130 °C) and 91.9% (160 °C) using BCMW as catalyst. BLMW exhibited 77.54% (130 °C) and 93.3% conversion at 160 °C.

Several authors reported first-order kinetics for esterification reactions [6,7,8,9,18,20]. With the plot of the graphs of ln (1 − conversion) versus time (Figure 11b), it was possible infer that there is a linear relationship between the consumption of DDPO and the time for all considered temperatures. The respective values of reaction constants (k) and correlation coefficients (R^2^) are shown in Figure 11b. The esterification reaction of DDPO is first-order in good agreement with literature reports.

From ln k versus 1/T plot of the graphics (Figure 11c), the values of the apparent activation energies (Ea) of the reaction [6,7,8,9,18,20] were calculated. Ea and their correlation coefficient (R^2^) are shown in Figure 11c. Reports in the literature clarify that low activation energies (ca. 10–15 kJ mol^–1^) indicate diffusion-controlled processes. For processes governed by truly chemical steps, generally the activation energy exceeds 25 kJ mol^–1^ [9,18,70]. In the present case, the activation energies observed for both catalysts were 30 and 32 kJ mol^−1^ for BLMW and BCMW, respectively, indicating that chemical steps ruled the esterification of DDPO (no diffusional issues are present in the reaction).

The apparent energy activation values observed for the esterification of DDPO with ethanol over BCMw and BLMW (30.0 and 32.2 KJ mol^−1^) are close to the activation energies (30.10 and 42.03 KJ mol^−1^) obtained by using a traditional H_2_SO_4_ homogeneous catalyst [48,66]. In another study involving clay-based catalysts, Oliveira et al. [8] found activation energy equivalent to 27.97 KJ mol^−1^ in the esterification of oleic acid; HPW supported on metakaolin prepared by Pires et al. [9], provides an activation energy of 19.78 KJ mol^−1^ for DDPO esterification with ethanol. Thus, the activation energies obtained in the present work are certainly comparable to those reported in the literature.

#### 3.9.6. Proposed Mechanism for the Esterification of DDPO

**Figure 12:**
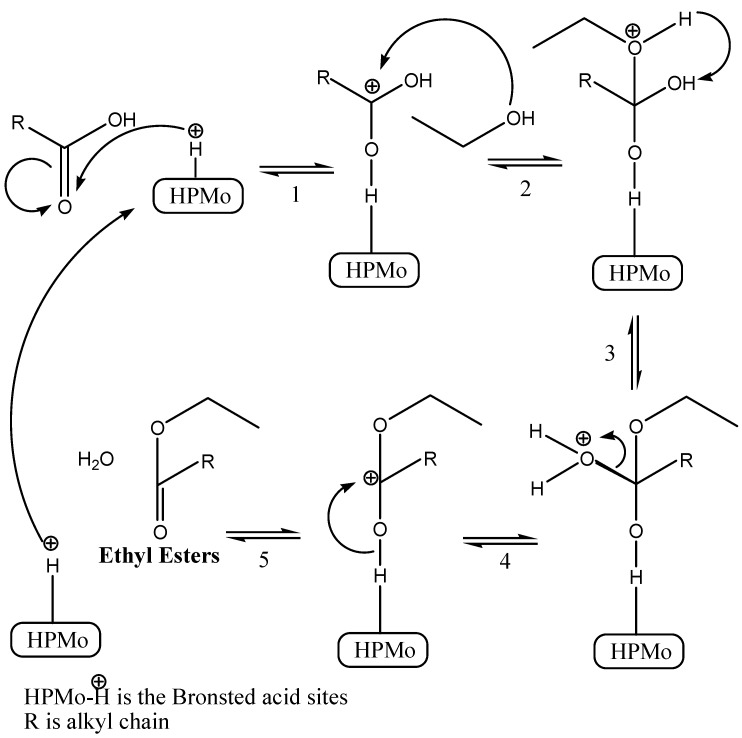
The reaction mechanism for esterification catalyzed by BLMW or BCMW.

The esterification of free fatty acids is an equilibrium-limited reaction. In order to overcome the equilibrium limitation, generally esterification of free fatty acids is carried out by taking alcohol (short-chain) in excess in order to favor the forward reaction [7,8,49]. The esterification of DDPO with ethanol is shown in Figure 12. The esterification reaction with short-chain alcohols follows an Eley–Rideal mechanism since the DDPO is only adsorbed on the surface of the catalyst, while the short alcohol directly reacts with it, without adsorption, from the gas phase; therefore the use of high proportions of alcohol to increase the autogenous pressure is necessary [5].

The active sites of HPMo perform the function of Brønsted acids as protons donor in the reaction [14,18,43,70]. Esterification occurs between free fatty acids (FFA) and alcohols. In the present case, free fatty acids are present in DDPO [9,39] and the selected alcohol was ethanol (EtOH). A proposed mechanism for this reaction on the BLMW and BCMW catalysts is proposed in Figure 12. 

In step 1, the interaction of carbonyl oxygen from the FFA with Brønsted acid sites from the surface of the catalysts forms a carbocation. In step 2, an unstable tetrahedral intermediate (I) is produced under nucleophilic attack, by one of the pairs of solitary electrons in ethanol oxygen, to carbocation. In step 3, the ethanol proton was rearranged and transferred to the carbonyl oxygen of the FFA. In step 4, a tetrahedral intermediate (II) is dehydrated and in step 5, the desorption occurs, where the proton of the tetrahedral Intermediate (II) is transferred to the HPMo present in the catalyst. Thus, the formation and diffusion of ethyl esters for the reactional medium occurs together with the restoration of the active sites of the catalyst (HPMo). As the esterification reaction is a reversible reaction, its inverse reaction of ester hydrolysis with water also occurs [9,18,42,70].

#### 3.9.7. Reuse of Catalysts

The reuse of catalysts (Name R) in the esterification of DDPO with ethanol was conducted in three consecutive cycles (R1, R2, R3), under the optimum conditions established for the reaction. The catalysts used, after each reaction cycle, were separated by filtration, washed with ethanol and then dried at 150 °C for 4 h to be reused. Table 5 shows that BCMW and BLMW catalysts are reusable, although exhibiting a certain activity loss during the esterification of DDPO. The catalysts possessed high DDPO conversions in the first reaction cycle as well as in the first cycle of reuse, with conversions close or above 90% (90.7% and 86.3% for BCMW, 93.3% and 89.1% with BLMW) and decreased from the second cycle of reuse. Nevertheless, the conversions obtained for the subsequent cycles are still significantly improved as compared to the blank reaction (in the absence of catalyst, 24%), indicating that the materials can be reused with good catalytic activities to esterify a complex fatty raw material (DDPO).

**Figure 13:**
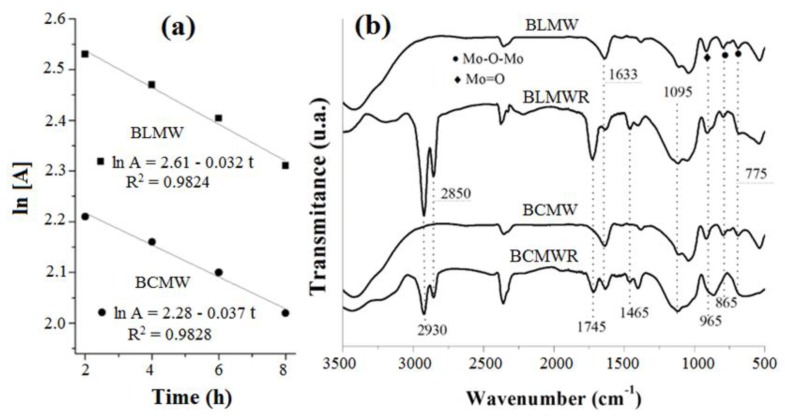
(**a**) Operational stability and plot of residual activity versus reaction time for the determination of inactivation constant and half-life; (**b**) FTIR spectrum for samples fresh BCMW, BLMW and before post-reuse BCMWR, BLMWR.

Turnover Frequency (TOF) and surface acidity of the catalysts was also reduced during the reuse stages. The results suggest that the reduction of surface acidity follows a first-order decay, following a linear graph Ln [A] versus time, where the deactivation constants and the respective linear regression coefficients (R^2^) can be obtained (Figure 13a). A half-life time of ca. 19–22 h was estimated for BCMW and BLMW catalysts. These values are in line with those reported in literature (23 h) for the synthesis of biodiesel on HPMo/Nb_2_O_5_ [14]. 

The catalytic activity reduction of catalysts may be related to factors such as loss of mass or even the pore blocking/destruction by remaining impurities even after the purification step and leaching of the active phase [9,14,20,42]. In order to verify the integrity of the catalysts after each cycle of reuse, materials were characterized by FTIR, XRD and EDS. While monitoring the presence of HPMo by UV-vis based on the absorption at 220 and 310 nm, assigned to the charge transfer ligand–metal (O^2^ → P) and charge transfer lingate metal (O^2^ → Mo^6+^) respectively [54]. The concentrations of the catalysts newly synthesized and after reaction were determined from the curve obtained (Figure 1a), which also determined leaching of the catalyst during reuse.

Fresh and reused catalysts were characterized by FTIR (Figure 13b). Compared to the FTIR spectrum of the fresh catalyst, new bands in 2931 and 2854 cm^−1^, corresponding, respectively, to the vibrations of the asymmetric and symmetrical stretch of the C-H bonds of the ethyl group were observed. Bands in 1742 cm^−1^, referring to the stretching of the C=O bonds, and in 1463 cm^−1^, attributed to the asymmetric deformation of the C-O bond of the ester group, appear after esterification, demonstrating the fixation of the esterification product in the reused catalysts [14]. Even after the catalysts have been used in the reactions, their FTIR spectra still contain the characteristic vibrations of HPMo close to 965, 865 and 775 cm^−1^ (Figure 13b). This indicates that HPM_O_ remains immobilized with its Keggin structure in the catalysts after the reactions [14,23]. 

**Figure 14:**
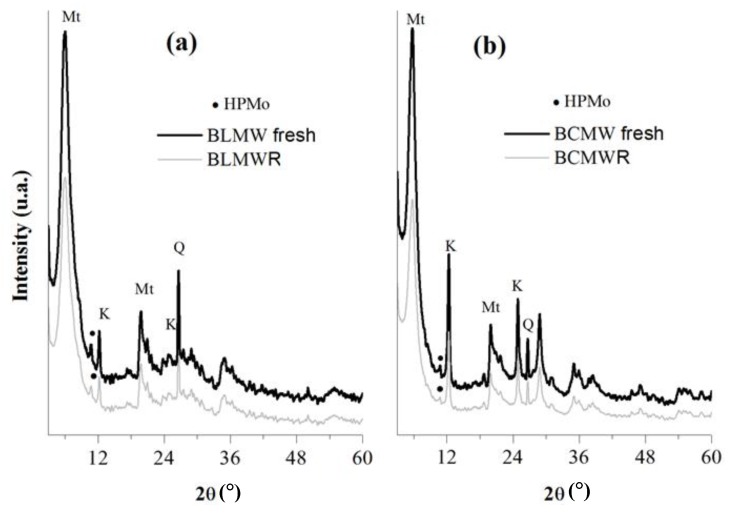
X-ray diffractograms of the samples fresh (BLMW and BCMW) and reused (BLMWR and BCMWR) catalysts. (**a**) BLMW fresh, BLMWR; (**b**) BCMW fresh, BCMWR.

In addition, the DRX diffractograms of the reused samples were compared with those of the fresh catalysts (Figure 14). After the reuse steps, almost no modification of the crystallographic patterns of the supported catalysts was evidenced. There was only a decrease in the intensity of the Mt, K and Qs diffraction signals of the reused catalysts, compared with the original precursors (BL and BC). These effects may be due to the adsorption of organic molecules on the surface or within the porous of the catalysts, which promoted the reduction of the size of the crystals and alter the intensity of the DRX peak [47], despite the reactions being conducted under harsh reaction conditions in terms of mechanical agitation and temperature [27], no leaching of Mo to the obtained ester phases was observed for the monitored samples. Therefore, by the results of XRD and FTIR, the catalysts showed good structural and thermal stability during the reaction process.

Leaching of the active supported species makes the catalyst unattractive and, therefore, it is necessary to study the leaching of HPMo from the solids, mostly related to the solubility of HPMo in ethanol [9]. HPMo has been reported to leach to the ethanolic phase during esterification [14,42]. By means of EDS (see Supplementary Material) and UV-vis analyses, percentage drops of Mo are observed in the catalysts in each reaction cycle (Table 5). Based on these analyses, it was demonstrated that the BLMW catalyst presented a leaching close to 2% in each reaction cycle. The literature reports that for a reaction to occur eminently in heterogeneous phase, leaching should not exceed 2.7% in each reactional cycle [20]. Thus, it was demoted that BCMW and BLMW solid catalysts can be reused, without significant losses of their activities.

Considering other catalyst studies which report washing with tert-Butyl alcohol and very high reaction times [14], leaching of heteropolyacid [5] and significant loss of activity [20], optimum catalysts prepared in this work show advantages including minumum leaching of active HPMo species, good recyclability with moderate loss of activity and simple regeneration in the absence of high temperature calcination (as reported by [21,68,69]).

## 4. Conclusions

The synthesized BLMW and BCMW solids exhibited excellent activities for the esterification of DDPO with ethanol, with values comparable to traditional homogeneous catalysts and higher than other heterogeneous catalysts. The prepared materials have good catalytic performances even after a very simple recycling process in three reuse cycles, without significant loss of their activities. FTIR and XRD results further demonstrated a strong interaction between HPMo and the support (BC and BL) even after anchoring followed by ethanol washing step as well as for recycling catalysts. The kinetic study established that the esterification of DDPO follows a first-order reaction. It was shown here that a green and sustainable process, using natural bentonite supported with HPMo, can be used and reused sometimes to transform DDPO, which is a byproduct of the oilchemical industry, in ethyl esters.

## Figures and Tables

**Table 1:** Chemical compositions of samples in percentage (%).

Sample	BC	BL	BCM	BLM	BCMW	BLMW
**O**	42.11	47.73	35.94	39.89	37.74	30.88
**Si**	36.55	28.39	27.29	22.92	28.44	28.79
**Al**	12.49	10.50	9.51	7.28	10.22	10.10
**Fe**	5.06	9.26	3.76	8.21	4.72	7.83
**Mg**	2.36	1.75	1.58	1.36	0.62	1.53
**K**	0.21	0.82	0.21	0.25	0.45	1.02
**Ca**	0.23	0.64	0.39	0.28	0.43	1.25
**Na**	0.79	0.42	0.90	0.91	0.98	0.62
**Ti**	0.20	0.88	0.20	0.50	0.85	0.36
**P**	-	-	0.08	0.07	0.054	0.065
**Mo**	-	-	18.15	17.72	15.01	16.97
**Total**	100	100	100	100	100	100

**Table 2:** Specific surface area, porous area, porous volume and surface acidity.

Catalyst	^a^ SSA (m^2^ g^−1^)	^b^ Sμ (m^2^ g^−1^)	^c^ V_p_ (cm ^3^ g^−1^)	^d^ D_p_ (nm)	mmol of H^+^ g^−1^
**HPMo**	-	-	-	-	20.7
**BC**	24	22	0.02	7.8	4.2
**BL**	43	22	0.04	7.1	5.0
**BCM**	<10	<10	-	-	10.7
**BCMW**	17	17	0.01	12.5	9.1
**BLM**	<10	<10	-	-	13.3
**BLMW**	23	23	0.02	18.1	12.5

^a^ SSA = BET specific surface area. ^b^ Sμ = porous area calculated by the BJH method. ^c^ V_P_ = porous volume calculated by the BJH method. ^d^ D_P_ = porous size calculated by the BJH method. HPMo = amount: 0.0439 g.

**Table 3:** Conversion and Turnover Frequency (TOF) data for raw materials and the fresh catalysts.

	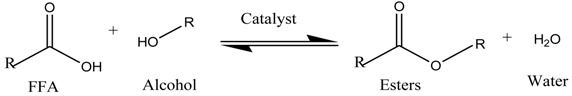			
Catalyst	Conv. (%) ^a^	Conv. (%) ^b^	TOF (min^−1^) ^c^	TOF (min^−1^) ^d^
**HPMo**	88.3	94.4	76	82
**BC**	25.3	27.5		
**BCM**	95.1	97.1	82	84
**BCMW**	90.7	93.3	105	108
**BL**	28.4	30.7		
**BLM**	94.4	95.3	82	83
**BLMW**	93.3	94.5	93	110

^a^ Conv. Conversion values for Esterification of the DDPO with ethanol (1:30), catalyst amount: 0.175 g (5%) after 2 h at 160 °C. ^b^ Conv. Conversion values for Esterification of the DDPO with methanol (1:30), catalyst amount: 0.175 g (5%) after 2 h at 160 °C. ^c^ TOF (Esterification of the DDPO with ethanol); ^d^ TOF (Esterification of the DDPO with methanol). Turnover frequency (TOF) is defined as the number of moles of DDPO converted per moles of active Mo species in the catalyst in unit time [42].

**Table 4:** Results of free fatty acid esterification (FFA) catalyzed by different solid acid catalysts.

Catalyst	Temperature (°C)	Time (h)	FFA	Alcohol	Mole Ratio	Conversion (%)	Reference
Al-MCM-41 Si/Al = 8	130	2	palmitic	Methanol	1:60	79	[6]
MF9S4	160	4	Oleic	Methanol	1:60	98.9	[7]
MF9S4	160	4	DDPO	Methanol	1:60	92.8	[39]
20%H_3_PW/ZrO_2_	100	4	Oleic	Ethanol	1:6	88	[21]
MF8S4M4W15	115	2/3	Oleic	Methanol	1:60	96.5	[8]
25%HPW/MK700	200	2	DDPO	Ethanol	1:10	83	[9]
MP-S-16 (15)	140	5	CKO	Methanol	1:8	82	[41]
HPMo/Nb_2_O_5_	210	4	macaw	Ethanol	1:90	99.7	[14]
AM41-2H-O	130	2	DDPO	Methanol	1:30	98	[49]
H_2_SO_4_	130	1	DDPO	Methanol	1:3	90	[48]
H_2_SO_4_	60	3	PFAD	Methanol	1:3	62	[66]
H_2_SO_4_	50	14	Oil Soapstock	Methanol	1:18	99.9	[67]
CrWO_2_	170	3	PFAD	Methanol	1:2	86	[68]
CrWTiO_2_	170	3	PFAD	Methanol	1:2	83	[69]
BLMW	160	2	DDPO	Ethanol	1:30	93.3	Present work
BCMW	160	2	DDPO	Ethanol	1:30	90.7	Present work

**Table 5:** Reuses of the catalysts under optimized reaction conditions.

Cycle	Mo (%)^a^	HPMo (mg)^b^	TOF (min^−1^)	Acidity (mmol H^+^g^−1^)^c^	Conversion (%)
**Blank**					<20
**BCMW**	15.01	31.5	105	9.2	90.7
**BCMWR 1**	13.88	30.2	104	8.6	86.3
**BCMWR 2**	12.25	28.2	101	8.1	79.0
**BCMWR 3**	12.11	27.5	98	7.5	73.1
**BLMW**	16.97	36.9	93	12.5	93.3
**BLMWR 1**	16.90	36.2	90	11.9	89.1
**BLMWR 2**	16.48	35.3	86	11.1	83.5
**BLMWR 3**	15.32	34.0	84	10.1	78.3

^a^ Determined by EDS. ^b^ Determined by UV-vis ^c^ by Boehm [56,57,58] method. R = Reuse.

## Supplementary Materials

The following are available online at https://www.mdpi.com/1996-1944/12/9/1431/s1, Figure S1: EDS spectra of (a) BC; (b) BCM; (c) BCMW; (d) BCMWR1; (e) BCMWR2; (f) BCMWR3; (g) BL; (h) BLM; (i) BLMW; (j) BLMWR1; (k) BLMWR2 (l) BLMWR3.
