# Supplementary material for: Bentonites Modified with Phosphomolybdic Heteropolyacid (HPMo) for Biowaste to Biofuel Production
Source: Materials (Basel). 2019 May 2;12(9):1431. doi: 10.3390/ma12091431 (PMC6539966; doi:10.3390/ma12091431)
Supplement: Supplementary file 1 [file materials-12-01431-s001.pdf]

materials

MDPI

Supplementary Material

Bentonites Modified with Phosphomolybdic Heteropolyacid (HPMo) for Biowaste to Biofuel Production

Alex de Nazaré de Oliveira [1,2,3], Marco Aurélio Barbosa de Lima [1], Luíza Helena de Oliveira Pires [1], Moisés Rosas da Silva [1], Patrícia Teresa Souza da Luz [4], Rômulo S. Angélica [5], Geraldo N. da Rocha Filho [1,2], Carlos Emmerson F. da Costa [1,2], Rafael Luque [6,7,]* and Luís Adriano Santos do Nascimento [1,2,]*

1 Laboratory of Catalysis and Oilchemistry, Federal University of Pará, Augusto Corrêa Street, Guamá, 66075-110 Belém, Pará, Brazil; alexoliveiraquimica@hotmail.com (A.d.N.d.O.); marbalim@hotmail.com (M.A.B.d.L.); lulenapires@hotmail.com (L.H.d.O.P.); moisesrosas06@hotmail.com (M.R.d.S.); narciso@ufpa.br (G.N.d.R.F.); emmerson@ufpa.br (C.E.F.d.C.)
2 Laboratory of Oils of the Amazon, Federal University of Pará, Perimetral Avenue, Guamá, 66075-750 Belém, Pará, Brazil
3 Department of Exact and Technologic Sciences, Federal Ubiversity of Amapá, Rod. Juscelino Kubitschek, km 02-Jardim Marco Zero, 68903-419 Macapá, Amapá, Brazil
4 Federal Institute of Education, Science and Technology of Pará, Campus Belém, Avenue Almirante Barroso, Marco, 66093-020 Belém, Pará, Brazil; pdaluz@yahoo.com
5 Laboratory of X-Ray Diffraction, Federal University of Pará, Augusto Corrêa Street, Guamá, 66075-110 Belém, Pará, Brazil; rsangelica@gmail.com
6 Department of Organic Chemistry, Universidad de Córdoba, Ctra Nnal IV-A, Km 396, E14014, Cordoba, Spain
7 Peoples Friendship University of Russia (RUDN University), 6 Miklukho-Maklaya str., 117198 Moscow, Russia
* Correspondence: q62alsor@uco.es (R.L.); adrlui1@yahoo.com.br (L.A.S.d.N.); Tel.: +55-91-98171-4947 (L.A.S.d.N);

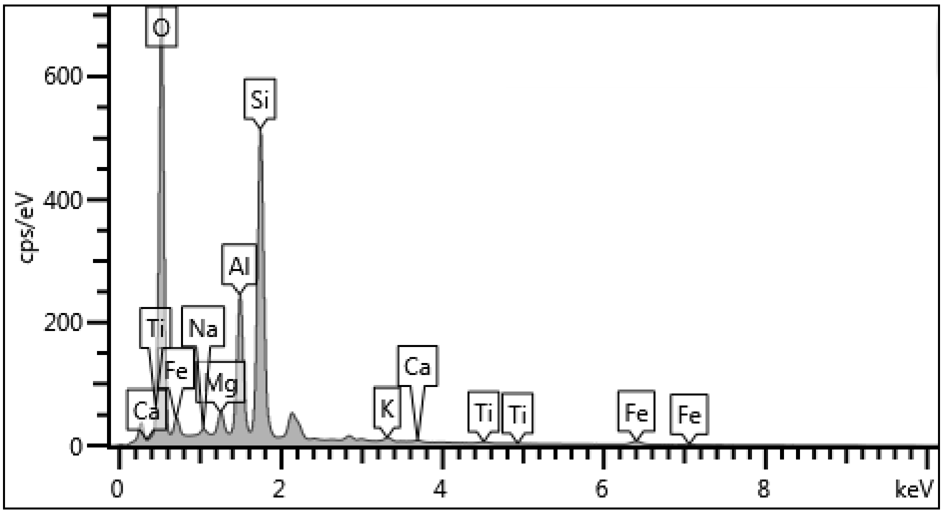

(a) BC

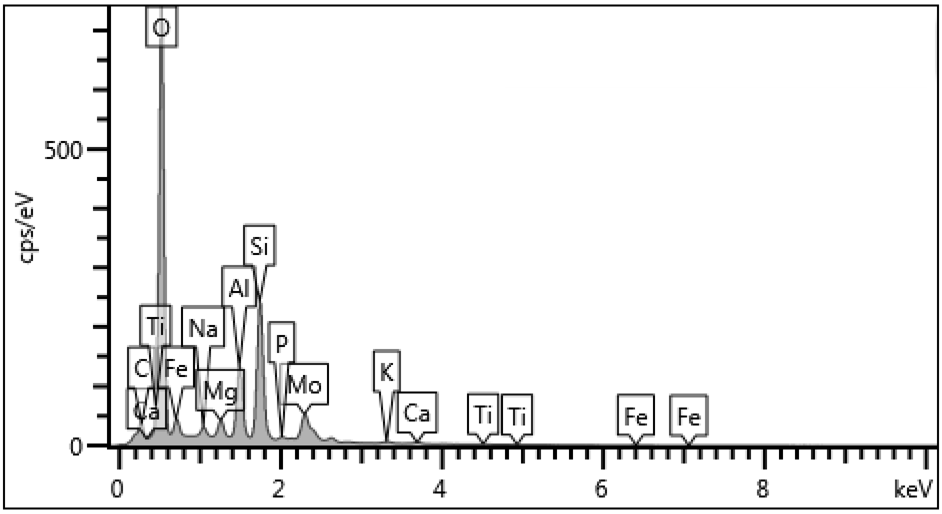

(b) BCM

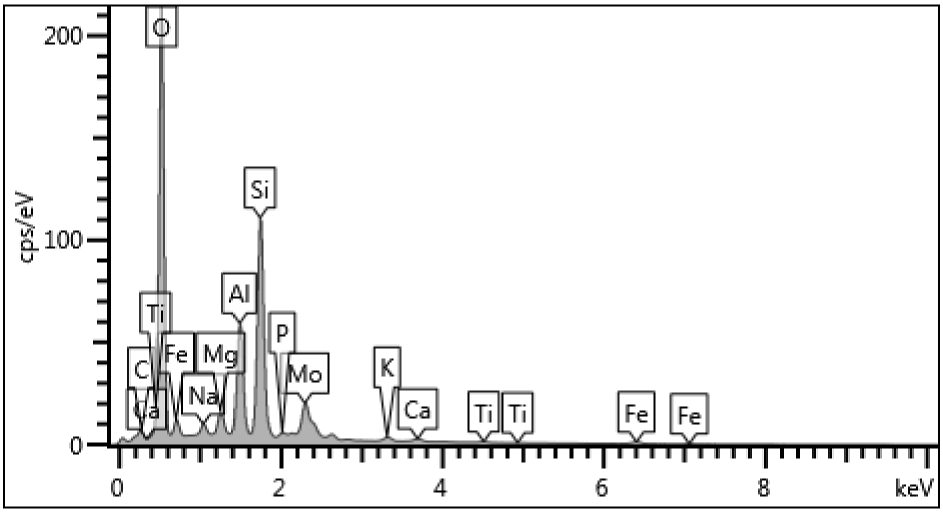

(c) BCMW

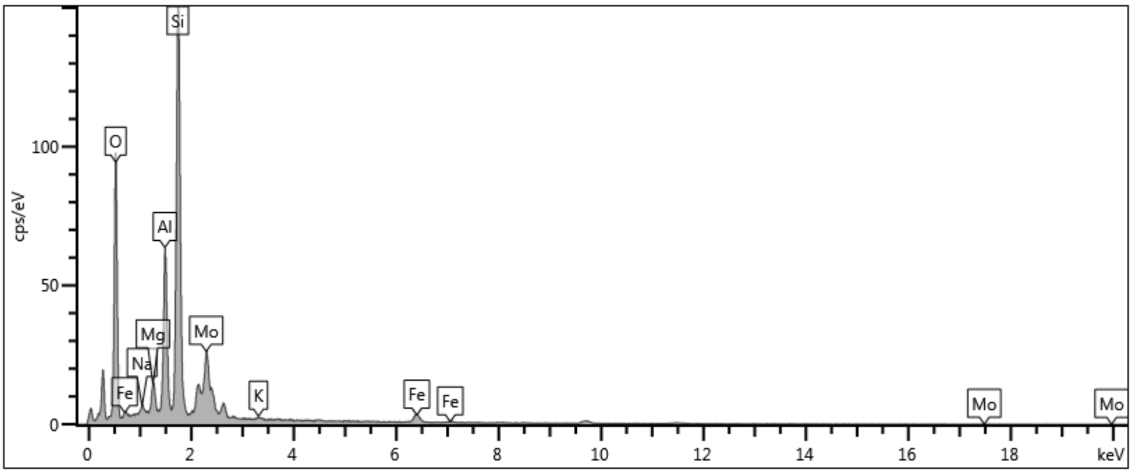

(d) BCMWR1

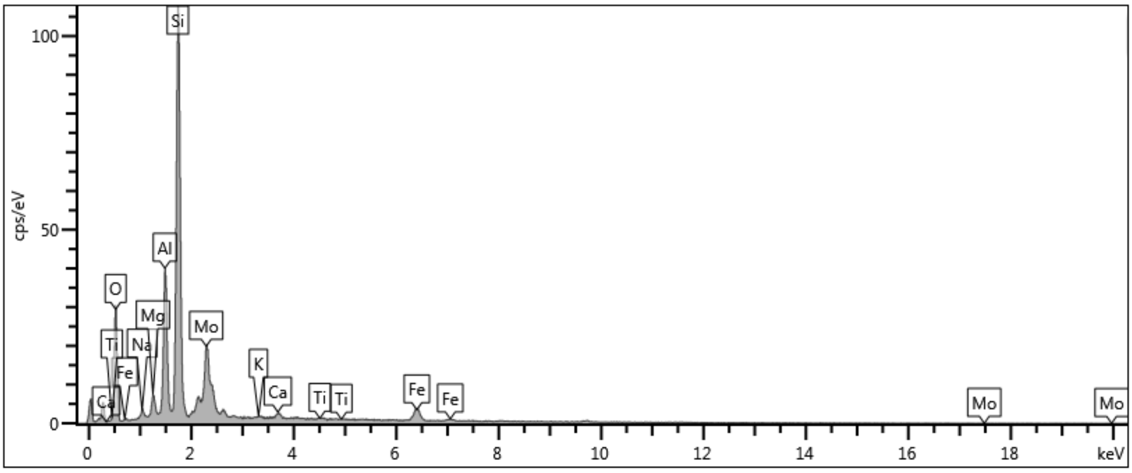

(e) BCMWR2

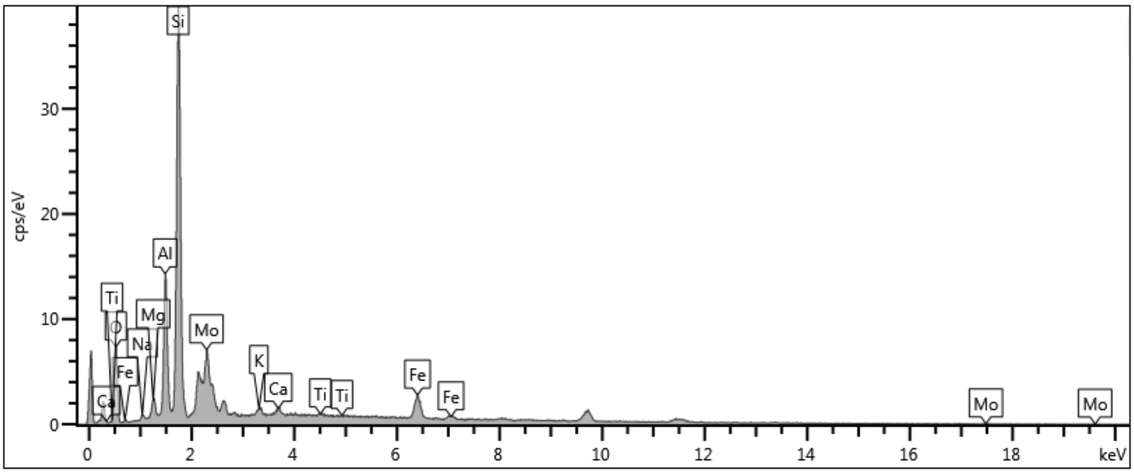

(f) BCMWR3

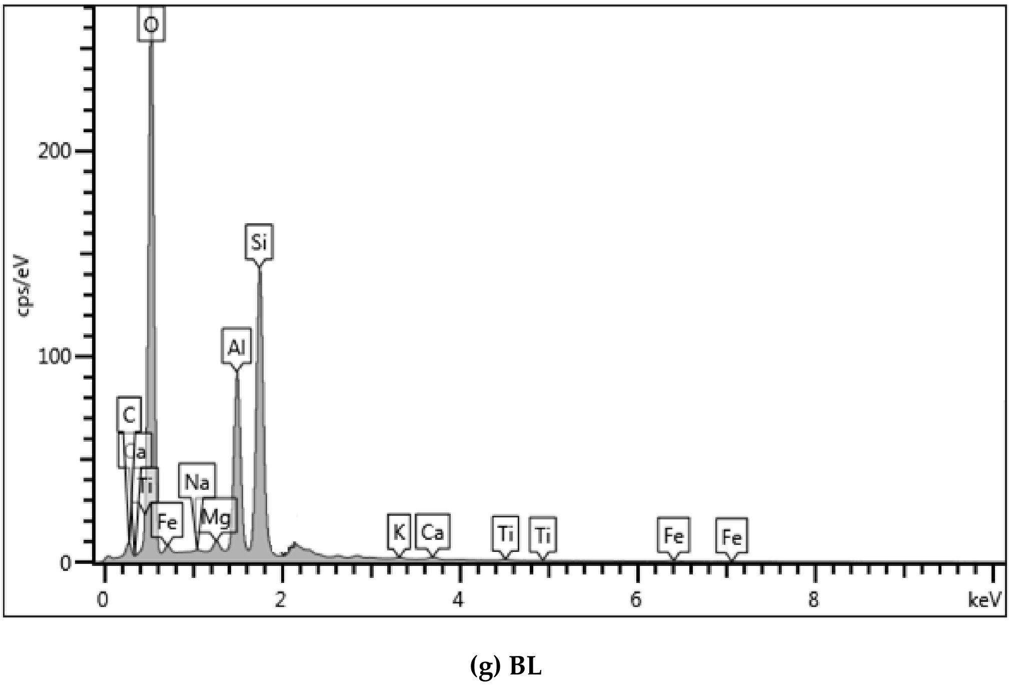

(g) BL

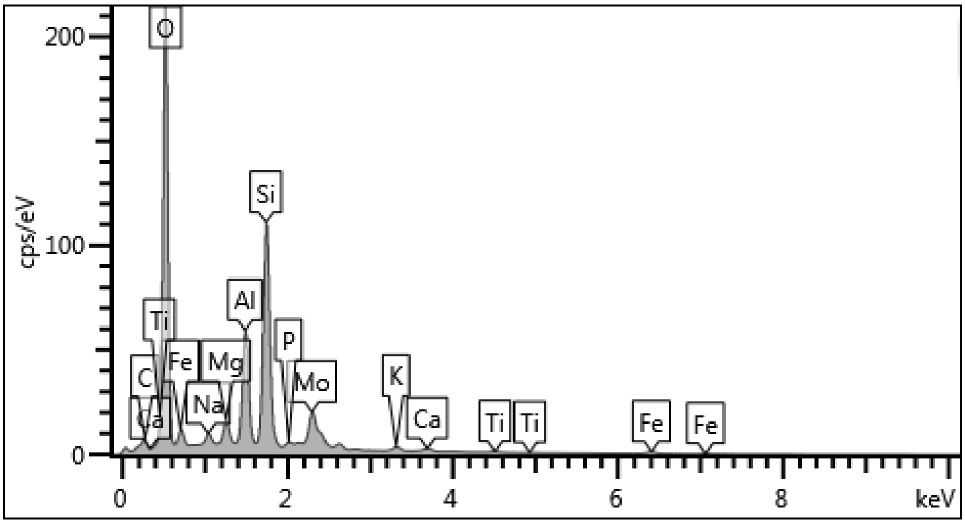

200
100
0
cps/eV
O
Si
Al
Ti
P
C
Fe
Mg
Mo
K
Na
Ca
Ca
Ti
Ti
Fe
Fe
0
2
4
6
8
keV

(h) BLM

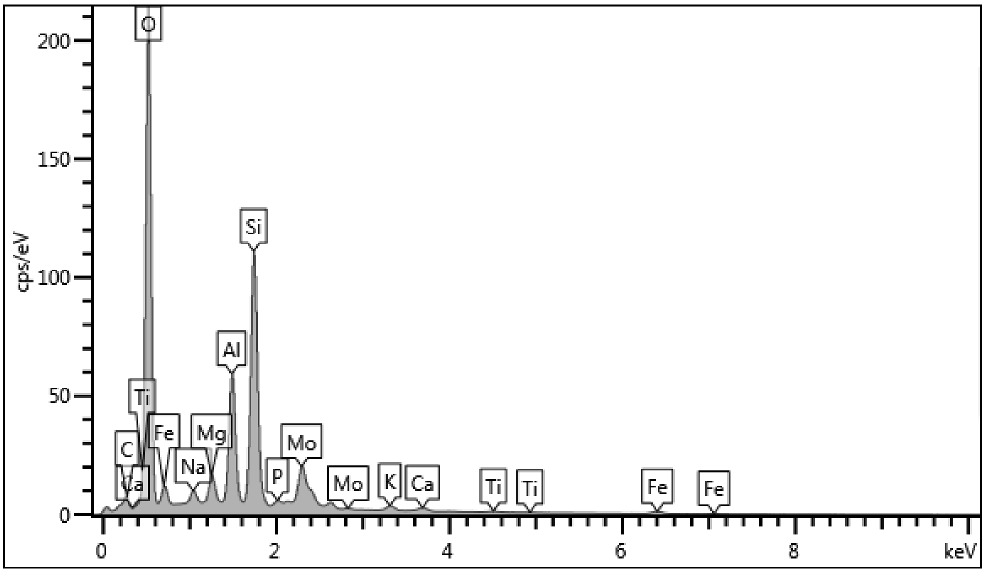

200
150
100
50
0
cps/eV
O
Si
Al
Ti
C
Fe
Mg
Mo
Na
P
Ca
Mo
K
Ca
Ti
Ti
Fe
Fe
0
2
4
6
8
keV

(i) BLMW

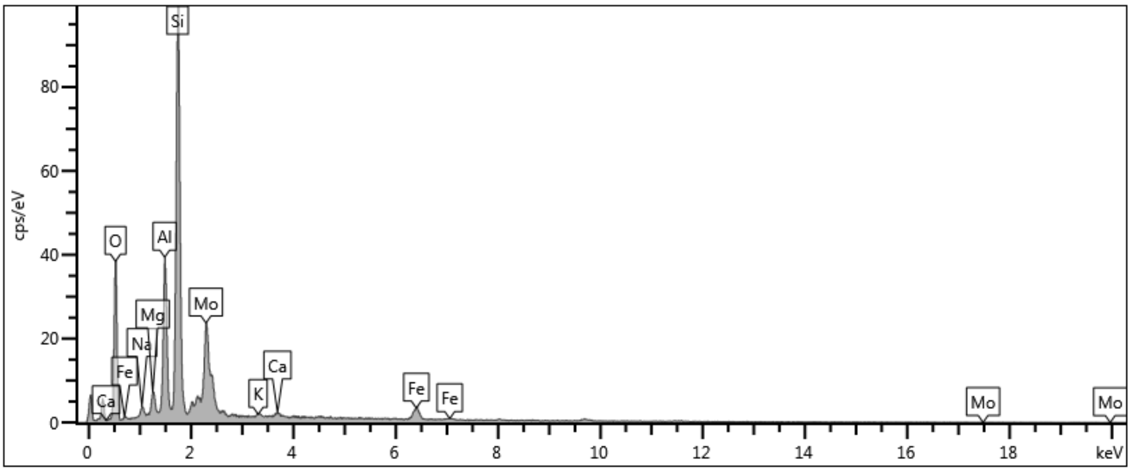

(j) BLMWR1

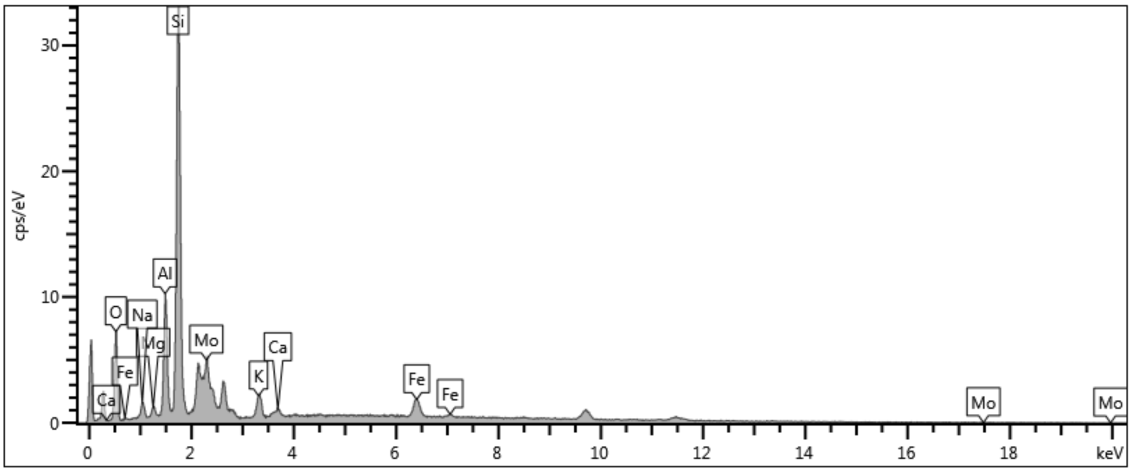

(k) BLMWR2

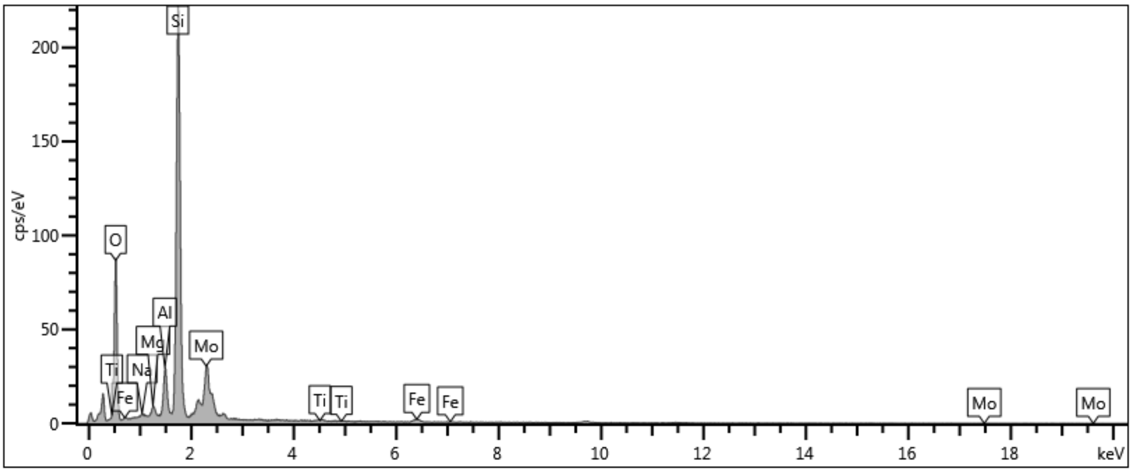

(l) BLMWR3

Figure S1. EDS spectra of (**a**) BC; (**b**) BCM; (**c**) BCMW; (**d**) BCMWR1; (**e**) BCMWR2; (**f**) BCMWR3; (**g**) BL; (**h**) BLM; (**i**) BLMW; (**j**) BLMWR1; (**k**) BLMWR2 (**l**) BLMWR3.

© 2019 by the authors. Submitted for possible open access publication under the terms and conditions of the Creative Commons Attribution (CC BY) license (http://creativecommons.org/licenses/by/4.0/).
